# PDX-derived organoids model in vivo drug response and secrete biomarkers

**DOI:** 10.1172/jci.insight.135544

**Published:** 2020-11-05

**Authors:** Ling Huang, Bruno Bockorny, Indranil Paul, Dipikaa Akshinthala, Pierre-Oliver Frappart, Omar Gandarilla, Arindam Bose, Veronica Sanchez-Gonzalez, Emily E. Rouse, Sylvain D. Lehoux, Nicole Pandell, Christine M. Lim, John G. Clohessy, Joseph Grossman, Raul Gonzalez, Sofia Perea Del Pino, George Daaboul, Mandeep S. Sawhney, Steven D. Freedman, Alexander Kleger, Richard D. Cummings, Andrew Emili, Lakshmi B. Muthuswamy, Manuel Hidalgo, Senthil K. Muthuswamy

**Affiliations:** 1 Cancer Center and; 2 Department of Medicine, Beth Israel Deaconess Medical Center, Harvard Medical School, Boston, Massachusetts, USA.; 3 Departments of Biology and Biochemistry, Boston University, Boston, Massachusetts, USA.; 4 Department of Internal Medicine I, University Hospital Ulm, Ulm, Germany.; 5 NanoView Biosciences, Boston, Massachusetts, USA.; 6 Department of Surgery and; 7 Department of Pathology, Beth Israel Deaconess Medical Center, Harvard Medical School, Boston, Massachusetts, USA.

**Keywords:** Oncology, Cancer, Glycobiology

## Abstract

Patient-derived organoid models are proving to be a powerful platform for both basic and translational studies. Here we conduct a methodical analysis of pancreatic ductal adenocarcinoma (PDAC) tumor organoid drug response in paired patient-derived xenograft (PDX) and PDX-derived organoid (PXO) models grown under WNT-free culture conditions. We report a specific relationship between area under the curve value of organoid drug dose response and in vivo tumor growth, irrespective of the drug treatment. In addition, we analyzed the glycome of PDX and PXO models and demonstrate that PXOs recapitulate the in vivo glycan landscape. In addition, we identify a core set of 57 N-glycans detected in all 10 models that represent 50%–94% of the relative abundance of all N-glycans detected in each of the models. Last, we developed a secreted biomarker discovery pipeline using media supernatant of organoid cultures and identified potentially new extracellular vesicle (EV) protein markers. We validated our findings using plasma samples from patients with PDAC, benign gastrointestinal diseases, and chronic pancreatitis and discovered that 4 EV proteins are potential circulating biomarkers for PDAC. Thus, we demonstrate the utility of organoid cultures to not only model in vivo drug responses but also serve as a powerful platform for discovering clinically actionable serologic biomarkers.

## Abstract 2

Pancreatic ductal adenocarcinoma tumor organoids are effective models for predicting in vivo drug response and discovering new biomarkers.

## Graphical abstract

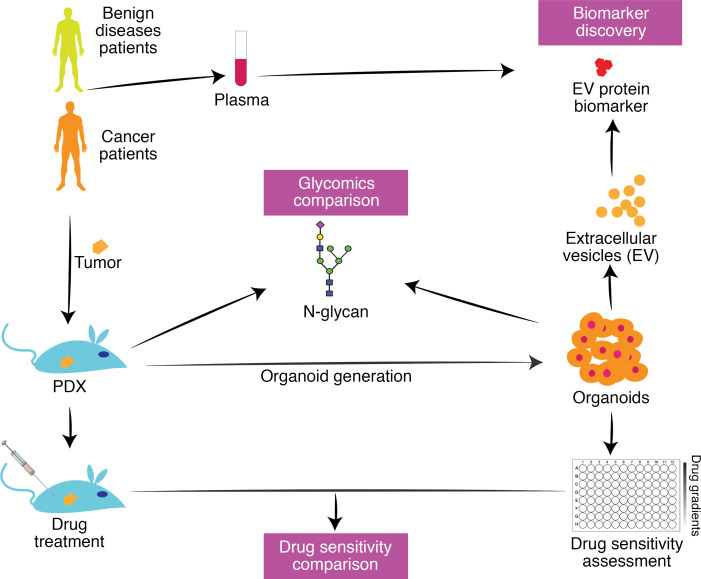

## Introduction

Translational cancer research has benefited significantly from the use of patient materials either for genomics analysis or for the generation of patient tumor-derived models such as xenograft (PDX) or organoid cultures (1). These patient-derived models frequently retain inter- and intrapatient variations of the disease, which is a significant advantage over the traditionally used immortalized cancer cell lines that suffer from genetic drift and variance from original patient tumors due to long-term maintenance in culture. PDX models are known to maintain multiple aspects of cancer traits and model therapeutic drug response in patients with more than 85% accuracy (2). Despite their high fidelity in modeling human cancer, routine use of PDX models for all aspects of translational research is impeded by their high cost and the extended time needed to conduct experiments. Furthermore, PDX models are not an ideal platform for large-scale studies aimed at screening multiple therapeutic drugs and their combinations because of high cost and length of time. Patient tumor-derived organoid (PDO) models are quickly evolving as a method to model a patient’s disease ex vivo. We and others have reported distinct methods to generate PDO models for pancreatic cancer using media containing serum or serum-free conditions with or without WNT ligands (3–6). Recent studies using the WNT-containing media point to the ability of PDO models to retain genetic and drug response properties when compared with patient responses retrospectively (7, 8). Seino et al. reported that WNT influenced the subtype of pancreatic ductal adenocarcinoma (PDAC) tumor organoids established in culture (5). Driehuis et al. further demonstrated that the influence of WNT in culture media on organoid phenotypes was through population selection (9). We reported that our WNT-free pancreas tumor organoid media (PTOM) was effective in retaining both the in vivo differentiation status of the patient tumor and the intratumor histological heterogeneity, demonstrating the potential to retain in vivo–relevant tumor biological traits in culture. To better understand the utility of organoids generated under PTOM conditions, we compared drug sensitivity in matched sets of PDX and PDX tumor-generated organoid (PXO) models and developed an analysis strategy that can be used to predict in vivo activity using in vitro drug response. In addition, we explored the potential to use organoid models for biomarker discovery by analyzing extracellular vesicles (EVs).

Both N- and O-glycosylation are significantly altered in malignant tissues, and such changes can profoundly affect protein function in multiple ways, including protein maturation, localization, folding, cell adhesion, protein trafficking, cell signaling, and immune response (10). In pancreatic cancer, CA19-9, a carbohydrate antigen, is known to promote activation of EGFR signaling and induce a pancreatitis phenotype and when combined with Kras^G12D^ resulted in the development of pancreatic cancer (11), demonstrating glycosylation changes are not just biomarkers but can function as drivers of the oncogenic process. However, glycosylation changes are rarely studied in patient-derived models, likely due to resource limitations associated with using PDX models. Here, we demonstrate that PXO cultures retain the complex glycosylation changes that are observed in PDX models, thus identifying organoid platforms as a powerful model for investigating cancer-associated changes in the glycome.

Early detection of pancreatic cancer is also an urgent need in oncology, and current methods to identify candidates for cancer biomarkers are limited (12). Identifying blood-based biomarkers, such as EV-associated proteins, that can distinguish cancer from benign diseases is frequently viewed as a needle-in-a-haystack challenge (13). Interestingly, employing EV nucleic acid cargo as a circulating biomarker source appears to be a superior diagnostic and monitoring test for PDAC (14). Using PDX models to discover EV-based protein biomarkers is a nontrivial task in part because of the presence of contaminating host factors. Here we demonstrate that media supernatants from PXO cultures can be used to find EV protein markers for PDAC. We further performed clinical validation of these markers in a small cohort of patients with benign pancreatic diseases, gastrointestinal (GI) diseases, or PDAC, demonstrating that these potentially novel markers were selective for PDAC and underscoring the potential of PXO as a pipeline for biomarker discovery.

## Results

### Tumor organoids retain genetic and phenotypic identity of tumors in vivo.

**Figure 1:**
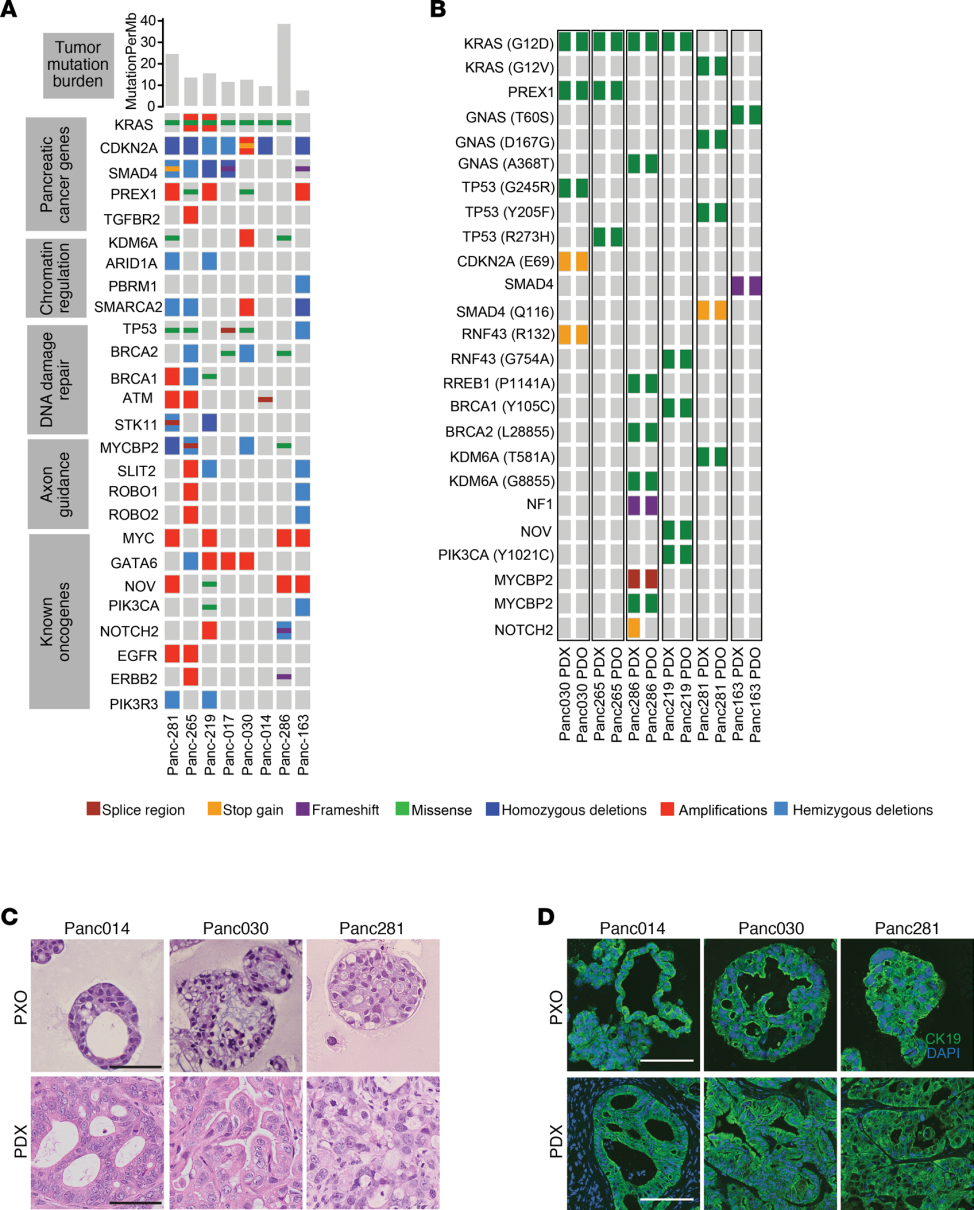
Genomic and histological features of tumor organoids and matched PDX tumors. (**A**) Genomic alterations in PDX tumors deduced by exome sequencing were used to calculate mutation burden (top bar graphs), and mutations corresponding to common pathways are shown. Color scheme used: green, missense mutation; purple, frameshift; yellow, stop gained; brown, splice region mutated; dark blue, homozygous deletion; light blue, hemizygous deletion; red, amplification. (**B**) Major oncogenic mutations in PDX tumors and matched PXOs. Color scheme for genomic alterations is coded as outlined in **A**. (**C**) H&E images of PXO and matched PDX tumors from 3 representative tumors. Scale bars: 50 μm. (**D**) Expression of pancreatic ductal cell marker cytokeratin 19 in PXO and matched PDX tumors. Scale bars: 50 μm.

To build a set of matched PDX and tumor organoid models, we identified 8 PDX models that have heterogeneous combination of alterations in the common pancreatic cancer oncogenes, such as *KRAS*, *TP53*, *CDKN2A*, *SMAD4*, *c-MYC*, *GATA6*, *ERBB2*, as well as other genetic alterations in SWI/SNF, DNA damage repair, and axon guidance pathways (15–17) as determined by exome sequencing analysis of the PDX tumors (Figure 1A). The models included 1 *KRAS^wt^*, 1 *KRAS^G12V^*, 1 *KRAS^Q61H^*, and 5 *KRAS^G12D^* (Figure 1A). All models were PDAC except Panc014, which was cholangiocarcinoma (see Supplemental Figure 1A; supplemental material available online with this article; https://doi.org/10.1172/jci.insight.135544DS1 for patient information). We generated organoids from those models using a method described previously (3). Comparison of exome sequences of 6 organoids and their matched PDX models revealed an overall concordance of genomic alterations, except 1 allele *NOTCH2* containing a stop codon not found in the corresponding PXO (Figure 1B). Twenty-four of the 25 allelic alterations of frequently mutated genes in PDAC matched between tumor organoids and PDX tumors, demonstrating that organoid culture grown in WNT-free medium maintained critical genomic features of the matched PDX tumors.

To determine if tumor organoids recapitulate the biology and architecture of the PDX models, we analyzed organoid sections by H&E stain and using a molecular marker (cytokeratin 19) that is expressed in ductal epithelia of the pancreas. PDX tumors had high epithelial contents with minor stromal components (Supplemental Figure 1B). Panc014 PDX tumors had glands predominantly cribriform in architecture, with focal regions of poorly differentiated morphology; Panc030 PDX tumors had glands that were confluent, with focal single cells; Panc281 PDX tumors had glands somewhat cribriform/confluent but still easily identifiable as glands, with focal poorly differentiated areas (Figure 1C). Tumor glands were consistently observed in all organoid cultures. Overall, the correlation of histopathological features between PDX and organoids was very apparent in Panc014 and Panc281, while in Panc030 it was less clear, demonstrating that organoid cultures mostly retain intermodel variations in histopathology. Tumor cells in organoids and PDX tumors expressed cytokeratin 19 (Figure 1D), demonstrating that cells in culture and PDX models retained pancreatic epithelial differentiation.

**Figure 2:**
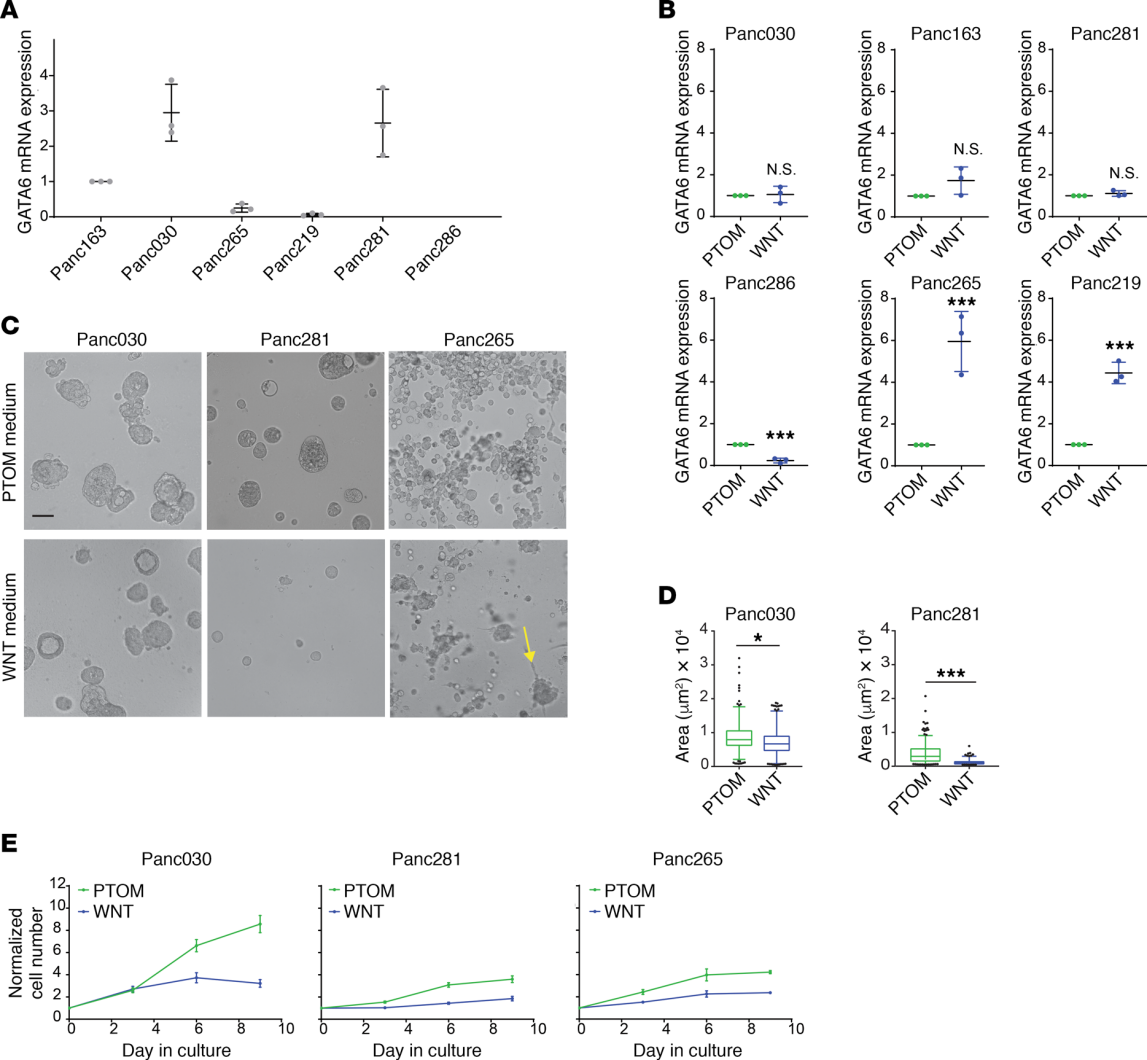
Differences in biology of organoids cultured in WNT-free and WNT-containing culture media. (**A**) Expression of *GATA6* mRNA in PXOs grown in PTOM. Scatter plots: bars represent maximal and minimal values; central lines represent mean values; dots represent results from independent experiments; *N* = 3. (**B**) Changes of *GATA6* mRNA expression in PXO grown in WNT-containing culture media, *N* = 3. (**C**) Phase contrast images of PXO (day 9 in culture) grown in WNT-free (PTOM) and WNT-containing (WNT) media. Scale bars: 100 μm. (**D**) Areas of PXO from Panc030 and Panc281 lines. Over 100 PXOs from 3 independent cultures were used for analysis. (**E**) Changes in cell number of PXO grown in different culture media. *N* = 3. Two-tailed *t* test was used to calculate statistical significance. *P* value indicators: N.S., *P* ≥ 0.05; *0.01 ≤ *P* < 0.05; ***P < 0.001.

We investigated if our PTOM conditions made an objective difference in organoid growth and differentiation status compared with WNT-containing media conditions previously reported (4). The Sato and Clevers laboratories reported that *GATA6* expression levels affect the growth of PDAC organoids in WNT-dependent and WNT-independent culture conditions, identifying *GATA6* as an indicator of WNT-regulated biology in PDAC organoid cultures (5, 9). Analysis of *GATA6* levels in the 6 models established in PTOM demonstrated that Panc030 and Panc281 expressed high levels of *GATA6* compared with the other models (Figure 2A). We next investigated whether PTOM and Clevers’ organoid media (referred to as WNT media) influenced *GATA6* expression and growth properties of PDAC organoids. Organoid lines established in our PTOM were switched to WNT media for 3 passages, then analyzed. Interestingly, 2 out of the 6 lines (Panc265, Panc219) showed a significant upregulation while 1 line (Panc286) had downregulation of *GATA6* expression in WNT media, demonstrating that presence of WNT can influence the differentiation status of organoids (Figure 2B). In addition to gene expression changes, organoids maintained in 2 media had different morphology (Figure 2C). Panc030 and Panc281 organoids maintained in PTOM were larger than organoids maintained in WNT media (Figure 2D). Panc265 organoids, which were derived from a highly metastatic tumor, exhibited grape-like structures with no observable large solid organoids in PTOM, while in WNT media we frequently observed large solid organoid (on average 3–4 structures per ×10 field) along with grape-shaped clusters (indicated by the yellow arrow in Figure 2C). Cell growth rates in all 3 lines were also higher in PTOM compared with cells grown in WNT media (Figure 2E). These results demonstrated that differentiation status and cell biology of organoids were differentially regulated in PTOM and WNT media. Although a head-to-head comparison between these culture conditions can provide important insights, in this study, we first focused our efforts on demonstrating the utility of PTOM organoids in modeling in vivo–relevant drug response and its use as a platform for identification of clinically actionable biomarkers.

### Concordance between PXO and PDX responses to therapeutic drugs.

Response to therapeutic drugs in PDX models is known to correlate well with patient responses (2). Recent studies using culture methods that used media developed by the Clevers/Tuveson groups demonstrate that drug responses observed in pancreatic PDO cultures reflected those observed in in vivo contexts (4, 7, 8). We investigated if organoids generated in our conditions, which lack WNT ligands, can phenocopy drug responses in PDX models. We reasoned that a successful outcome would help demonstrate the utility of the organoid models grown in cost-effective conditions as a scalable platform for preclinical and translational research efforts aimed at finding and validating experimental drugs and drug combinations.

**Figure 3:**
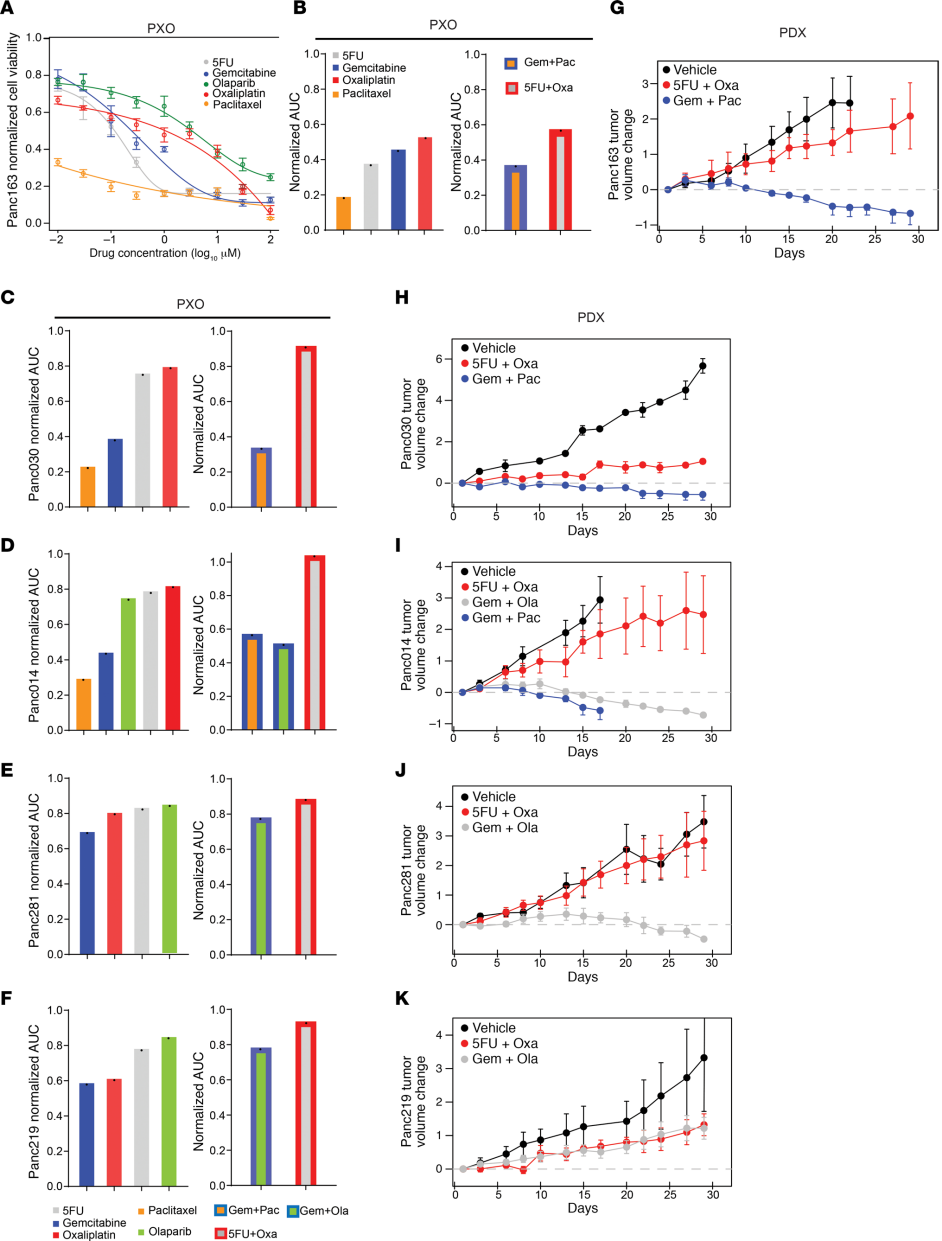
Concordance between PXO and PDX responses to therapeutic drugs. (**A**) Changes in Panc163 PXO survival in response to single-agent treatments (*N* = 6). (**B**) Normalized AUC values for Panc163 PXO survival in response to single-agent (*N* = 6) or combinational treatments (*N* = 3). Each bar represents 1 AUC value (indicated by 1 black dot in each bar) calculated from the fitted curve per treatment. Normalized AUC values for PXO in response to single-agent (*N* = 6) or combinational treatments (*N* = 3) for (**C**) Panc030, (**D**) Panc014, (**E**) Panc281, and (**F**) Panc219. Change in tumor volume in PDX models (*N* ≥ 3): (**G**) Panc163, (**H**) Panc030, (**I**) Panc014, (**J**) Panc281, and (**K**) Panc219.

To best simulate PXO application in a clinically relevant setting, we compared PXO and PDX responses to standard-of-care chemotherapies used to treat patients with PDAC, namely, gemcitabine, paclitaxel, oxaliplatin, 5-fluorouracil (5FU), and olaparib. Five models, Panc163, Panc030, Panc014, Panc281, and Panc219, which have genomic alterations in pathways typically dysregulated in pancreatic cancer, were selected for these studies (Figure 1A). Although patients with PDAC received combination drug treatments, determining response to drug combinations in culture is a challenging task because of factors such as drug dosing, length of treatment, and the order of administration. In this regard, recent studies by Palmer and Sorger (18) demonstrate that patient-to-patient variations and independent drug action are sufficient to explain the efficacy of drug combinations in the clinic and that the clinical outcome of a combination can be correlated to the most effective single agent in the drug combination. In this study, we tested the response to single agents and combinations in PXO models by measuring cell death after 96 hours of drug exposure. For single-drug treatments, we tested a broad concentration range over 5 logs (10 nM to 100 μM, Figure 3A). With the exception of 5FU, maximal doses used in combination treatments were capped at peak plasma concentrations reported in patients (Supplemental Figure 2A) because testing higher doses would not have clinical significance. In addition, the ratios of the drugs used in the combination were modeled after ratios typically used in the clinic. To quantitatively compare responses to different treatments, we calculated the area under the curve (AUC) values for responses to each drug or drug combinations. For Panc163, paclitaxel treatment was most effective, while oxaliplatin was the least effective (Figure 3B). AUC for responses to combination treatments showed similar outcomes as in single-reagent treatment, with a paclitaxel-containing combination more effective than 5FU/oxaliplatin (Figure 3B). In all models tested, the most effective combination treatment was comparable to the effect observed for the most effective single-agent response in the combination (Supplemental Figure 2C; Figure 3, C–F). We did not observe additive or synergistic effects for these drug combinations in these PXO models. Treatment of PXOs with toxins, puromycin (2mg/mL)/cycloheximide (25 μM), induced over 70%–95% cell death, demonstrating that the cell death differences between organoid lines were not due to the difference in drug permeability (data not shown).

PDX mouse models with subcutaneously transplanted tumors were treated with drugs following the schedules listed in Supplemental Figure 2B. Consistent with PXO responses for Panc163 and Panc030, gemcitabine/paclitaxel was more effective in inhibiting tumor growth than 5FU/oxaliplatin in vivo (Figure 3, G and H). For Panc014, both gemcitabine/olaparib and gemcitabine/paclitaxel were effective in suppressing tumor growth compared with 5FU/oxaliplatin (Figure 3I), consistent with the observation that gemcitabine and paclitaxel were effective single agents and combinations in PXO studies. For Panc281 gemcitabine/olaparib was also more effective than 5FU/oxaliplatin in both PXO and PDX (Figure 3J). For Panc219, responses to gemcitabine and oxaliplatin were comparable in PXO (Figure 3F), and drug treatments on PDX also showed similar responses (Figure 3K). Thus, in all 5 models, we observed clear concordance between drug sensitivity ranking in PXO and the matched PDX tumors.

### PXO AUC values can predict in vivo drug response.

**Figure 4:**
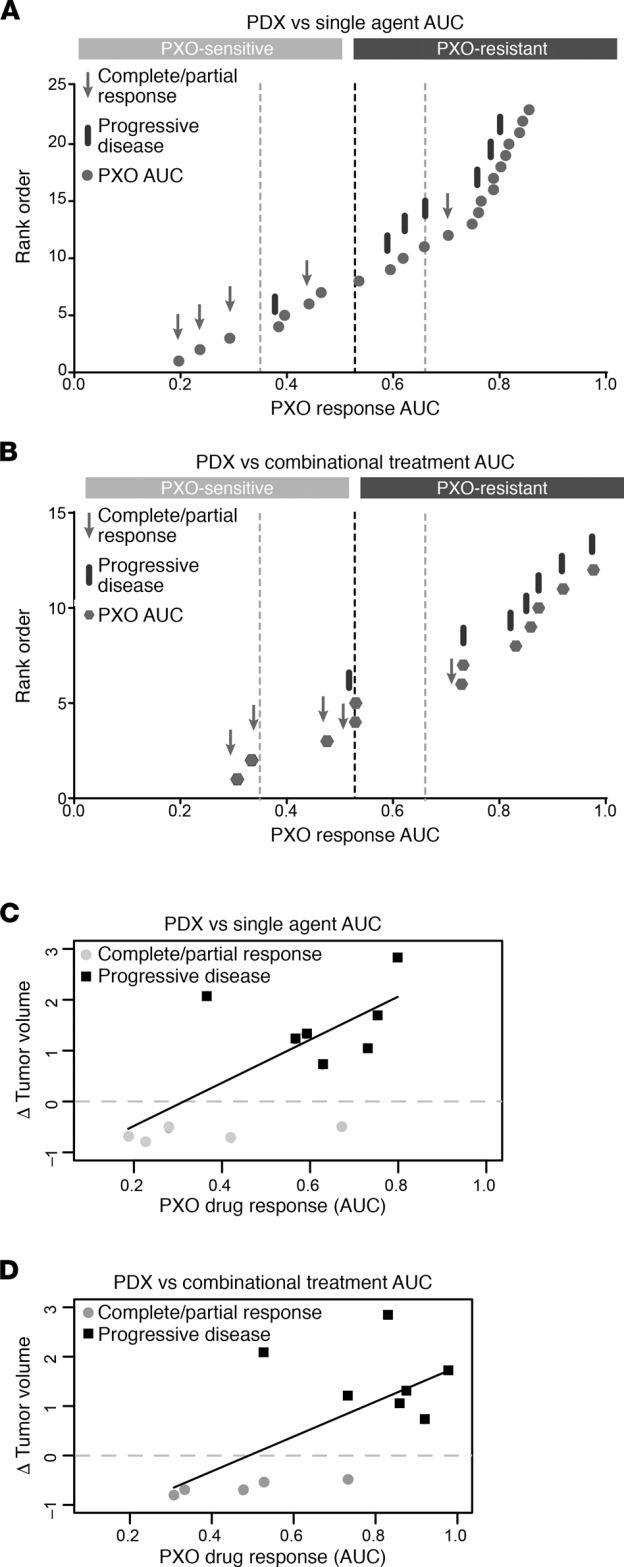
PXO AUC values can predict in vivo drug response. (**A**) Jenks Natural Break analysis of AUC values for PXO drug responses to single agents plotted as rank order versus normalized AUC; dashed lines represent thresholds for separation of groups calculated by Jenks analysis. For each drug combination tested in PDX models, the component agent with lowest AUC was compared with PDX responses: arrows refer to the PDX models with PR or CR (responder), and bars correspond to PD (nonresponder). (**B**) Jenks Natural Break analysis on PXO responses to combinational treatments as represented by normalized AUC values. Dashed lines represent thresholds for separation of groups calculated by Jenks analysis. Arrows refer to matched PDX models that showed response to the drug combination, and bars refer to matched PDX models that did not show response to the drug combination. (**C** and **D**) Linear regression fit to determine correlation between PXO AUCs and PDX tumor volume changes. Gray dots, PDX responders; black squares, PDX nonresponders.

We next investigated if the PXO AUC values can be used to predict in vivo drug response. The PDX responses were segregated as progressive disease (PD), partial response (PR), or complete response (CR) using a modified PDX RECIST criteria (see Supplemental Methods for detailed information) developed by The Jackson Laboratory (19) (Table 1 and Supplemental Figure 3A). The 24 normalized AUC values for PXO responses to single agents or 12 normalized AUC values for PXO responses to drug combinations were classified using the Jenks Natural Breaks algorithm, which identifies boundaries by minimizing intragroup variance and maximizing intergroup variance (Figure 4, A and B, vertical dashed lines). The AUC values were separated into 4 classes, the minimum number needed to observe the optimal classification of the data based on goodness-of-fit analysis (Supplemental Figure 3B). For the single-agent responses, the AUC values corresponding to individual PXO models were depicted as gray dots, and responses to in vivo treatments were identified as black bars representing PD and gray arrows representing PR or CR values (Figure 4A). The annotation of AUC values with the corresponding in vivo responses demonstrated that the Jenks break of 0.52 (segregation break, the dark dashed line) segregated the AUC values to match in vivo disease control (PR or CR) and PD groups with the highest consistency. This segregation of PXO single-agent AUC values demonstrated that PXO can be classified as “sensitive” to a drug combination in vivo if any one of the drugs in the combination regimen yielded an AUC less than 0.52 and “resistant” only if all the drugs in the regimen yielded an AUC at least 0.52. Four of 5 organoid treatments correctly classified PDX responders, and 6 of 7 organoid treatments correctly classified PDX nonresponders. The 2 mismatched groups were 5FU/oxaliplatin treatment on Panc163 and gemcitabine/olaparib treatment on Panc281. Although it is not clear why single PXO responses in Panc281 and Panc163 did not predict in vivo response, it is possible microenvironmental and genetic factors may play a role.

PXO response to combination treatments also segregated into sensitive and resistant groups at the segregation breakpoint AUC value of 0.52 (Figure 4B). PXO response to combination treatments also predicted in vivo response in 10 out of 12 times. To further understand if single-agent response and combination differ in the ability to correlate with in vivo response, we analyzed the correlation coefficient between PXO AUC and tumor growth inhibition (TGI) index of PDX tumors. Single agents and combination AUC values had comparable *R*^2^ values 0.34 and 0.42, respectively, by linear regression fit analysis (Figure 4, C and D), suggesting that using response to single agents to predict sensitivity to drug combinations will be a simple and reliable approach when using the drugs and combinations tested in this study. Thus, our data demonstrated that PXO responses to single-agent chemotherapeutic drug predict in vivo responses to combination chemotherapies.

### Organoids are effective in retaining the complex and specific glycosylation changes observed in tumors in vivo.

Like the genome of cancer cells, glycosylation of cellular proteins is known to undergo significant and varying alteration in cancer cells (20), which can have dramatic impacts on protein function, including its expression levels, stability, and localization (10). N-glycan and O-glycan profiles are known to vary among pancreatic cancer cells (21). Because glycosylated proteins are widely used as biomarkers and as therapeutic targets (22) and recent studies have demonstrated the ability of increased glycosylation to drive tumorigenesis in the pancreas (11), developing a better understanding of glycosylation changes using patient-derived models would be of significant advantage to the field.

**Figure 5:**
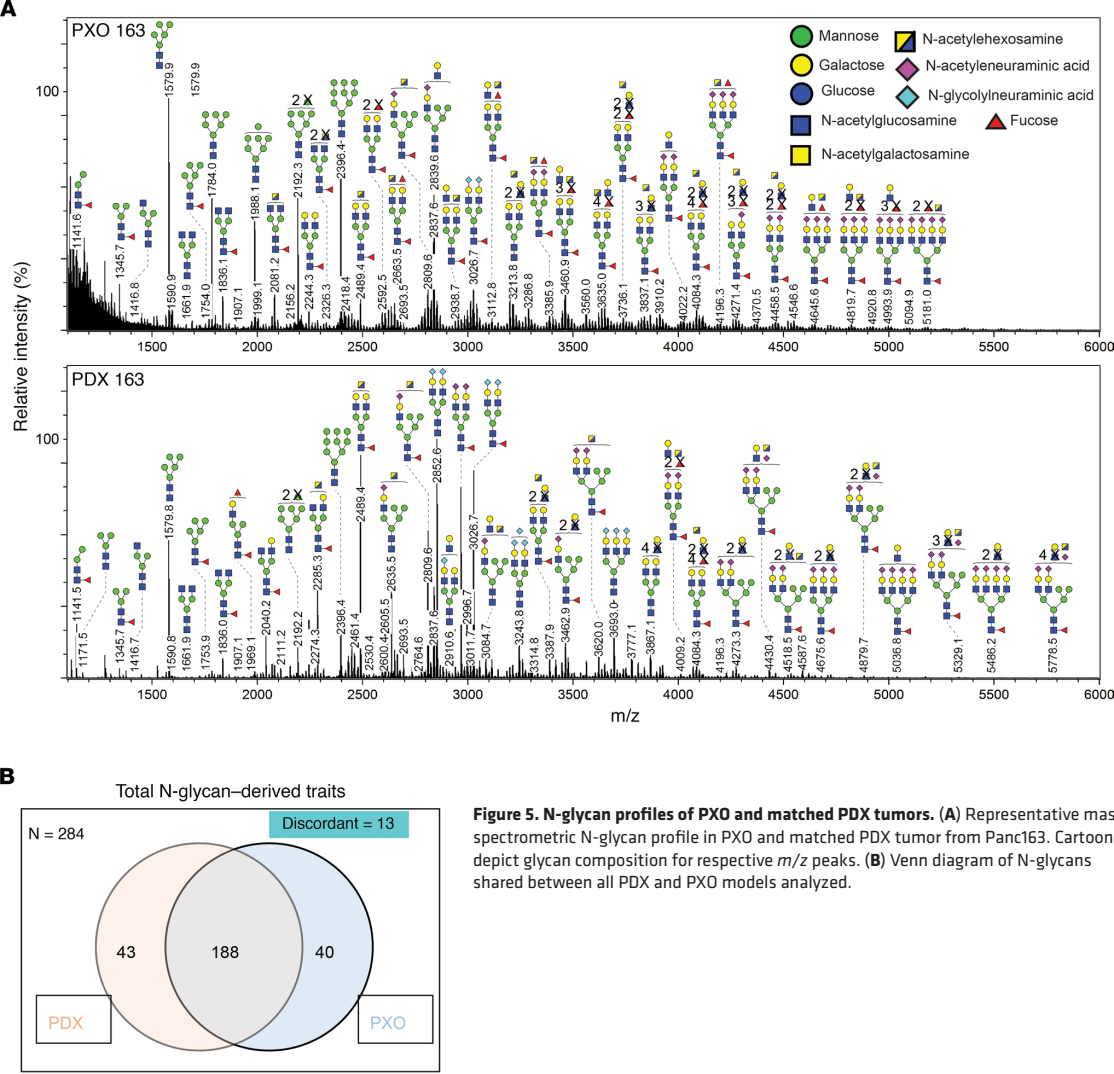
N-glycan profiles of PXO and matched PDX tumors. (**A**) Representative mass spectrometric N-glycan profile in PXO and matched PDX tumor from Panc163. Cartoons depict glycan composition for respective *m/z* peaks. (**B**) Venn diagram of N-glycans shared between all PDX and PXO models analyzed.

To determine if organoids can serve as a platform to understand glycosylation in patient-derived tumor models, we compared the N-glycan profiles of matched PDX and PXO models. Five pairs of PDX and PXOs were lyophilized and digested with trypsin then with PNGase F to release N-glycans, and the permethylated glycans were analyzed by matrix-assisted laser desorption ionization–time of flight (MALDI-TOF) mass spectrometry. Representative N-glycan profiles for a PDX-PXO pair are shown in Figure 5A and Supplemental Figure 4 with a few glycan masses annotated to demonstrate the overall similarity in the spectra between PDX and PXO samples. By analyzing 10 samples (5 PDX and 5 PXO), we identified a total of 284 N-glycan masses and predicted structures with each observed in a minimum of 1 sample. Interestingly 188 (66%) of them were shared by both PDX and PXO, and about 15% were unique to PDX or PXO (Figure 5B).

**Figure 6:**
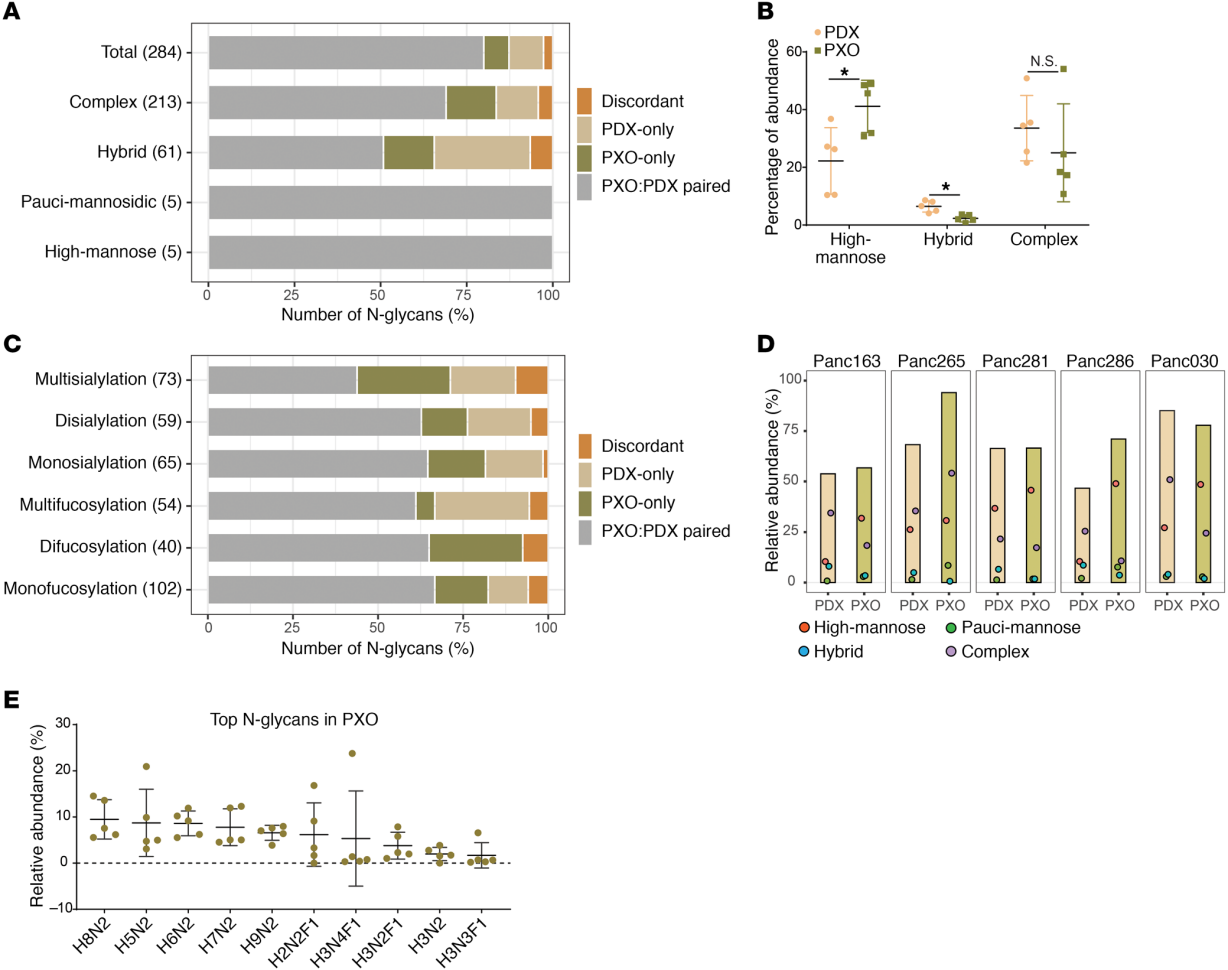
Comparison of N-glycan subtypes and abundance in PXO and PDX. (**A**) Major N-glycan classes and their occurrence in all PXO and matched PDX models analyzed. The *y* axis identifies the different classes of glycans, the numbers represent numbers of N-glycan in each class, and the *x* axis shows percentage of glycans with different distribution patterns. Paired, glycans identified in both matched PDX and PXO models; discordant, glycans identified in PDX and PXO from different tumors; PDX only, glycans identified only in PDX tumors; PXO only, glycans identified only in PXO. (**B**) Average relative abundance of 3 major N-glycan classes in PDX and PXO models; mean values and 95% confidence interval are indicated. (**C**) Numbers on *y* axis refer to numbers of the subgroups corresponding to varying degrees of sialylation or fucosylation, and *x* axis shows percentage of glycans with different distribution patterns. Chart format is the same as in **A**. (**D**) Relative abundance of the 57 common N-glycans in PDX or PXO samples. Colored dots indicate abundance of glycans in each class. Red, high mannose; green, pauci-mannose; blue, hybrid; purple, complex. (**E**) Distribution of top N-glycans in PXO. H, hexose; F, fucose; N, *N*-acetylglucosamine. Mean values and 95% confidence interval range are shown.

To determine if there are differences between PDX and PXO models in the subtypes of glycan modification, we first classified N-glycans into 4 major subtypes: (a) high-mannose: 5 to 9 mannose moieties are attached to the core, (b) pauci-mannose: less than or equal to 3 mannoses are attached to the core, (c) complex: where antennae are initiated by *N*-acetylglucosamine, and (d) hybrid: where mannose residues and 1 or 2 antennae are attached to the core (10). Among the 284 N-glycans, the complex subtype represented 87%–90%, followed by hybrid and high-mannose subtypes (Figure 6A) with concordance between PDX and PDO greater than discordant in all subtypes. To better understand the composition of subtypes, we estimated the relative abundance of each subtype in relation to the total glycan signal detected in PDX or PXO samples. The relative abundance of complex and high-mannose subtype was greater than the hybrid subtype in both PDX and PXO glycan profiles (Figure 6B). Interestingly, the relative abundance of complex glycans was similar between PDX and PXOs (Figure 6B). Thus, PXOs not only retain the diversity of different types of glycan but also retain the relative abundance of glycans similar to that observed in matched PDX models.

In cancer, the glycan subtypes display significant aberrations in sialylation or fucosylation due to changes in the expression of glycosyltransferases and fucosyltransferases (20). We classified the derived traits of N-glycans with varying degree of sialylation or fucosylation and observed a high degree of overlap between PDX and PXO samples modified by sialylation and fucosylation (Figure 6C), demonstrating that the most common cancer-associated alterations in glycosylation are also retained in PXO models as present in vivo PDX models.

When N-glycan profiles were analyzed in samples individually, PDX models on average had 143 ± 21 glycans, and PXO models on average had 138 ± 8.9 glycans, demonstrating that not all 284 glycans were observed in every sample analyzed. Fifty-seven N-glycans were present in all 10 samples. Interestingly, these recurrent 57 N-glycans (5 high-mannose, 3 pauci-mannose, 7 hybrid, and 42 complex) collectively represented 53% to 94% of total N-glycans observed in these samples, demonstrating that these 57 N-glycans dominate the N-glycan landscape in PDAC samples. Notably, the relative abundance of these 57 glycans was comparable between matched PDX and PXO models analyzed (Figure 6D). This unexpected observation raises the possibility that PDAC samples have a shared glycan signature, suggesting the conservation of underlying mechanisms of glycosylation. Organoids offer great opportunities to study tumor-relevant changes in glycobiology.

Looking at the average relative abundance in PXO models, we find that the top 10 N-glycans (Figure 6E) collectively contributed to approximately 60% of total abundance. Among these, there were 5 high-mannose glycans (H8N2, H5N2, H6N2, H7N2, H9N2), consistent with previous studies reporting increased levels of high-mannose glycans in pancreatic tumor regions, compared with normal tissues, detected by MALDI imaging mass spectrometry (23, 24) or by lectin probing (25). Four other glycans (H3N4F1, H3N2F1, H2N2F1, and H3N3F1) contained Lewis-X epitope (H(1,4)-Fα(1,3)-N) that was increased in PDAC and colon tumor tissues compared with normal (26, 27), demonstrating that PXO models retained glycosylation changes previously reported to be associated with PDAC. It is worth noting that there are over 110 proteins carrying a significant number of Lewis-X glycan epitopes (28), including those playing functional roles in PDAC, such as cathepsin D, collagens, laminin, LIF receptor, and KRAS (29–31). This finding further demonstrates that our PXO organoid cultures can serve as great tools for both the discovery and validation of glycosylation and their relevance to pancreatic cancer progression.

### Organoids as a discovery platform for blood-based biomarkers in patients with PDAC.

**Figure 7:**
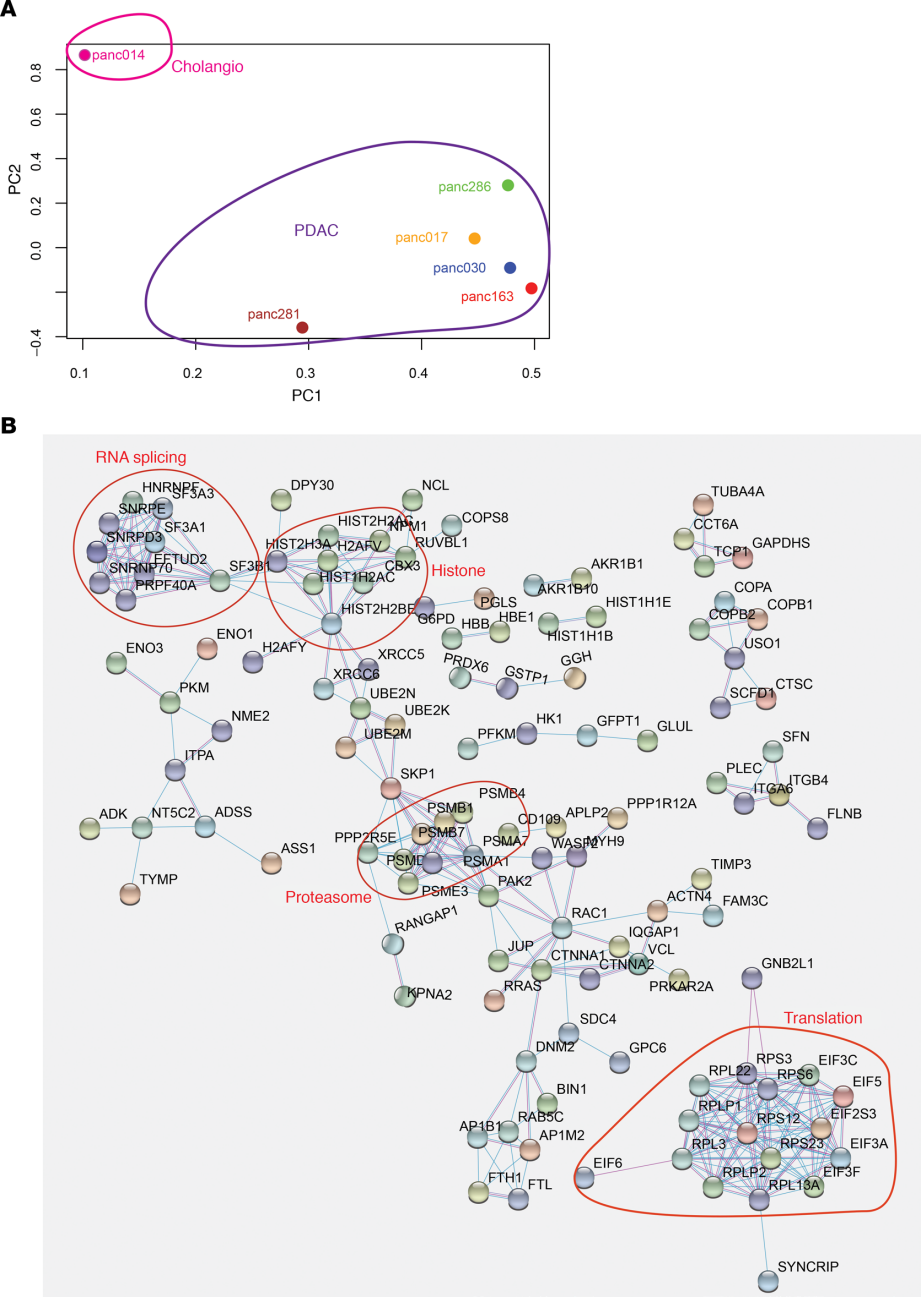
Identification of EV-associated proteins enriched in tumor organoid media. (**A**) PCA of EV-associated proteins identified in culture media of 6 PXO lines. (**B**) Functional clustering of EV proteins enriched in PXO supernatant-derived EV.

There is a significant need for diagnostic biomarkers in PDAC. One promising approach involves identification of secreted EVs in the blood of patients to differentiate patients with PDAC from patients who are disease free or with benign GI diseases. Several studies have attempted to identify EV-associated proteins using conventional cell lines in culture and validate them in blood samples from PDAC patients and healthy controls (32–36). It is not known whether organoid cultures can be used to discover clinically significant, secreted biomarkers. Typically, EV identification effort using cells in culture requires a large amount of media supernatant (0.1–1.0 L), which would be a technical challenge when using organoid cultures. To overcome this barrier, we optimized a vesicle enrichment method to concentrate vesicles from 4.0 mL of organoid media supernatant and subjected them to liquid chromatography-tandem mass spectrometry (LC-MS/MS). To differentiate cancer-associated EVs from those secreted by normal human pancreatic epithelial cells, we used media from our human embryonic stem cell–derived exocrine pancreas organoids (3). Among the 1465 proteins identified, 241 proteins were at least 2-fold higher in tumor organoid EVs compared with exocrine organoids and expressed in at least 4 out of the 6 tumor organoid lines (Supplemental Table 1). Principal components analysis (PCA) showed that the models differed from each other in the EV proteome. Interestingly, Panc014 segregated away from the rest of the samples (Figure 7A), consistent with fact that Pac014 was derived from a cholangiocarcinoma whereas all other models originated from PDAC, demonstrating our ability to detect distinct EV proteomic profiles among patient-derived models. In addition, it also highlights the fact that the culture/media conditions used to generate and maintain these organoid models do not induce neutralization of phenotypes but are effective in retaining interpatient heterogeneity in tumor biology.

To expand our understanding of the molecules present in EV, we used the STRING Database to analyze the 241 proteins identified. These proteins clustered into functional groups, including RNA splicing, histone/chromatin, proteasome, and translation, in addition to vesicles containing proteins involved in cytoskeleton regulation, cell adhesion, and membrane trafficking (Figure 7B). These observations are consistent with previous reports of proteins present in exosomes and other extracellular vehicles (32–36).

**Figure 8:**
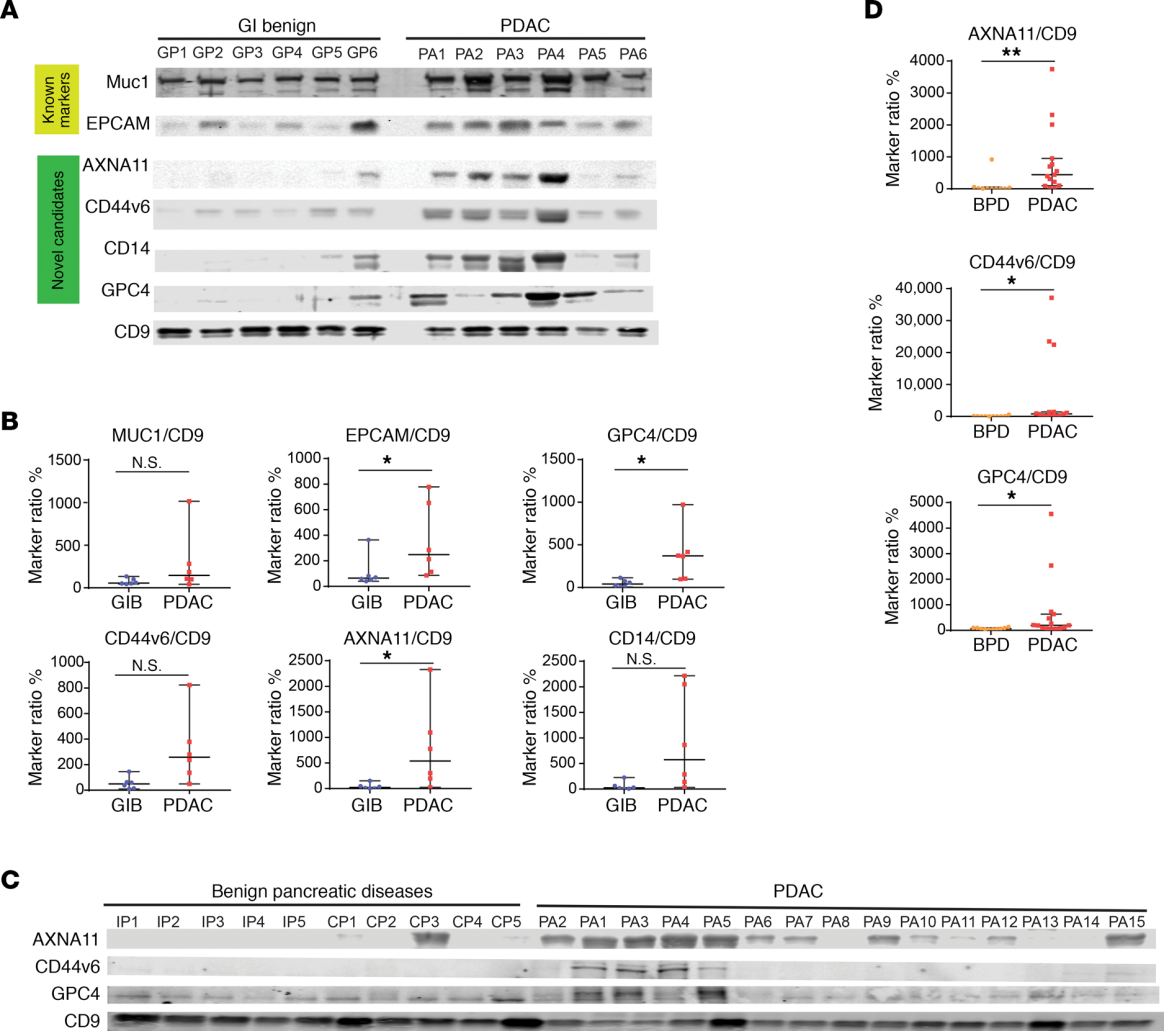
Validation of EV-associated proteins as biomarker candidates in patient plasma. (**A**) Immunoblot detection of EV protein markers in 15 μL plasma from patients with benign GI diseases or PDAC (PA). (**B**) Quantification of protein levels detected in the immunoblot shown in panel **C**. GIB, benign gastrointestinal diseases. EV marker signals were normalized to CD9 signals, then rescaled to median values (set as 100) of each marker. Median values and 95% confidence interval are shown. (**C**) Immunoblot detection of EV markers in 5 μL plasma from patients with pancreatic diseases. IPMN, intraductal papillary mucosal neoplasms; CP, chronic pancreatitis. PA, PDAC. (**D**) Relative signals of EV markers in plasma from patients with pancreatic diseases detected in the immunoblots shown in panel **C**. BPD, benign pancreatic diseases. Median values and 95% confidence interval are shown. Kolmogorov-Smirnov test was used to calculate statistical significance. *P* value indicators: N.S., *P* ≥ 0.05; *0.01 ≤ *P* < 0.05; **0.001 ≤ *P* < 0.01.

We further investigated whether a subset of EV proteins identified in organoid cultures could be detected in plasma from PDAC patients. We selected 5 markers: CD44 and GPC4 were enriched in EVs from PDAC and cholangiocarcinoma organoids, while VGLUT2, CD14, and annexin A11 were enriched in EVs from PDAC organoids but not cholangiocarcinoma organoids. Blood from 6 subjects with PDAC and 6 benign GI diseases were collected following an institutionally approved protocol. Patients’ ages ranged from 37 to 75 in the PDAC group and from 25 to 81 in the GI group (Supplemental Table 2). All PDAC patients were treatment naive, with stage I to stage IV cancer, and their CA19-9 values ranged from 3 to 6707 U/mL. EVs from plasma samples were purified using ExoQuick Ultra (SystemBio). We selected 3 EV markers, MUC1, EGFR, and EPCAM, previously known to be associated with PDAC (32, 33, 36), and 5 potentially novel markers from our study for analysis in protein lysates of purified EVs (Figure 8A and Supplemental Figure 5). Each lane had protein lysates of EVs from 15 μL of patient plasma. We were able to reliably detect MUC1 and ECAM in EVs from patient plasma, which showed a tendency to be enriched in PDAC patients, consistent with previous reports (32, 33, 36). To our surprise, 5 out of 5 of the new markers were detected in patients EVs with high sensitivity. Furthermore, among the 5 markers, annexin A11 (ANXA11), CD44v6, CD14, and GPC4 were enriched in the EV from patients with PDAC compared with the signal in patients with benign GI disease. This was not due to differences in the levels of EV isolated because the amount of CD9, a pan-EV marker, was similar across patients (Figure 8A). Interestingly, although PDAC patient BIDPA3 (PA3) had at least 30 times higher CA19-9 levels than patients PA4, PA5, and PA6 (Supplemental Table 2), the levels of the pan-EV marker and 4 PDAC candidate markers were not significantly different (Figure 8A), demonstrating a lack of association between CA19-9 levels and the enrichment of the EV markers. To quantitatively compare protein markers between the GI benign group and patients with PDAC, signals of each protein marker were normalized to standard EV protein CD9 and rescaled to respective median values as shown in Figure 8B (see Methods section for details). Normalized marker signals of EPCAM, GPC4, and ANXA11 were significantly higher (*P* < 0.05) in the PDAC patient group than the GI benign group.

To challenge the potential of EV markers CD14, GPC4, ANXA11, and CD44v6 in detecting PDAC patients, we analyzed their levels in a larger cohort of patients with PDAC and underlying pancreatic diseases: chronic pancreatitis and intraductal papillary mucinous neoplasm (IPMN) (Figure 8C). EVs used in each lane equaled 5.0 μL of plasma. We were not able to detect CD14 in this analysis probably because we used 3 times less plasma compared with the amount used in Figure 8A (15 μL plasma). We detected clear signals for ANXA11 in PDAC patient EVs but rarely in EVs from patients with benign pancreatic diseases. GPC4 signals were detected in both the PDAC group and the benign pancreatic disease group. Quantitative analysis showed that normalized signals of ANXA11, CD44v6, and GPC4 were significantly different from the benign group, with ANXA11 being the most promising marker candidate (Figure 8D).

## Discussion

Organoid cultures have garnered significant interest as a research tool in cancer biology. However, the strategies of applying organoid cultures in translational and clinical studies have not been well developed. Recent efforts provide strong support for the ability of PDX models to be efficient in predicting drug response in the clinic (2) and PDO models grown under WNT-containing culture conditions to be correlated with drug responses observed in PDX models (37). Seino et al. has reported that organoids dependent on WNT activation typically have reduced GATA6 expression (5). Driehuis et al. reported that when organoids can be generated from both WNT-containing and WNT-removed media, their gene expression patterns are highly similar in these 2 media conditions, suggesting the effects of WNT are likely to have selective impacts on tumor cell growth (9). In this report, we showed that organoids maintained in our PTOM grew faster than organoids maintained in WNT media. In addition, 2 out of 6 organoid lines upregulated *GATA6* expression when grown in WNT media, suggesting that presence or absence of WNT will affect the differentiation status of PDAC organoids. Although a detailed comparative analysis of PTOM and WNT media will be useful for the community, the goal of this study was to investigate the utility of organoids in PTOM for translational research.

Using WNT-free culture conditions, we methodically analyzed and modeled the PXO drug response and matched PDX in vivo response data. The analysis revealed a potentially novel method to classify AUCs as assessed by organoid drug dose response profiles to predict in vivo disease control. We find that sensitivity assessed for single agents is sufficient to predict response to drugs involving the agent, which is consistent with the model Palmer and Sorger (18) proposed and has implications for coclinical application of organoid sensitivity–based personalization of drug treatment for cancer patients. The ability to effectively classify in vivo responses to drug combinations based on sensitivities to single agents also has important implications for translational and clinical efforts where excluding an ineffective drug or drug combinations can be effectively used to avoid unnecessary toxicities in PDAC patients undergoing complex and aggressive treatment regimens with drug combinations.

Changes in protein N-glycosylation and O-glycosylation can profoundly impact protein maturation, expression, localization, and posttranslational modifications and impact its functions, such as ligand binding and signaling (10). In addition, aberrant glycosylation could also generate antigens that serve as biomarkers for cancer detection. In fact, the approved pancreatic cancer marker CA19-9 is a glycan antigen (38). Despite the broad and critical role glycosylation can play in cancer, little effort is being placed in understanding glycosylation changes in patient-derived models of cancer. A recent study using PDX models of human high-grade serous ovarian cancer demonstrated the feasibility to identify glycoproteins from PDX tumors that can be validated in the serum of ovarian cancer patients (39). In this study, we observed abundant high-mannose glycans in PXO models, which was consistent with findings in PDAC patient tumor tissues (23, 24). Increases in abundance of the complex type of glycans and fucosylation and sialyation have been associated with cancer (21). Consistently in both PDX and PXO models, complex glycans represent the most frequent type of modification, including fucosylated and sialyated glycans. Furthermore, we made an unexpected observation that among all the glycans identified, there is a core set of 57 N-glycans that are present in all patient-derived models we analyzed, and these 57 glycans collectively represent 50%–94% of the relative abundance of N-glycans, suggesting that they dominate the glycan landscape in PDAC. Profiling of glycomic alterations in patients with cancer has been a great challenge as MS-based methods require a significant amount of tissues, making them infeasible for primary tumor samples obtained during biopsy or even surgical procedures. Here, we demonstrate that tumor organoids established from PDX tumors can conveniently provide sufficient materials for such studies. In addition, organoids also have unique advantages for investigating the functional relevance of glycomic alterations because certain human glycosylation pathways are absent in mouse cells (40).

Identification of better blood-based biomarkers for pancreatic cancer diagnosis and disease monitoring is an urgent clinical need (13). We demonstrate a likely novel approach to use PXO models to discover diagnostic biomarkers by developing an organoid-based pipeline to identify EV proteins enriched in tumor media supernatant with subsequent validation in clinical samples. Previous EV proteomic discovery efforts relied on laborious techniques to isolate EV, typically requiring large amounts of supernatant from cancer cell lines (41). We demonstrate that small amounts of media from PXO culture supernatants can be used to purify EV, allowing for biomarker discovery studies. Proteomic profiles of EVs from PDAC PXOs and cholangiocarcinoma PXO clustered differently in PCA. We would need to analyze additional samples to know if the method distinguishes PDAC from cholangiocarcinoma; however, the results presented here show that our culture condition does not neutralize EV-related biology in organoids. In this effort, we identified EV protein markers that were not detected in previous studies using PDAC cell lines. When validated in patient plasma EVs, all 5 protein markers were detected, and 4 of them were enriched in the blood of PDAC as compared with subjects with benign GI diseases. Identification of markers that accurately distinguish pancreatic cancer from chronic pancreatitis and IPMN is a more challenging feat. More importantly we have shown that ANXA11 and GPC4 signals were significantly higher in plasma of PDAC patients as compared with subjects with benign GI diseases (*P* < 0.05, Figure 8B). These results strongly suggested that organoid culture can be a powerful platform to identify EV markers selective to PDAC compared with patients with benign pancreatic diseases. Aside from the potential role as diagnostic biomarkers, these markers could also provide insight into tumor biology. For example, annexin A11, a phospholipid-binding protein, regulates exocytosis and cytokinesis (42); CD44v6 is a CD44 splicing isoform frequently associated with tumor progression (43); CD14 is a monocyte-associated surface protein, which was recently shown to be expressed in cancer cells and to regulate the tumor microenvironment (44); VGLUT2 is a glutamate transporter; and GPC4 is a cell surface sulfate proteoglycan that regulates insulin and WNT pathways (45, 46). Further validation studies in an independent cohort of patients are needed to confirm the sensitivity and specificity of each marker and to determine whether combinations of multiple markers are needed to achieve enough diagnostic performance and enable the successful application of an EV-based biomarker in the clinic.

Our findings not only demonstrate the utility of organoids in our WNT-free conditions for translational research but also highlight an opportunity for exploiting the large collection of PDX models available in the scientific community by generating matched sets of PDX and PXO models to accelerate translational research. Our results also generate a road map for using PXO models as a powerful platform for studying glycosylation changes in cancer biology and for rapidly and effectively identifying biomarkers that can be translated to the clinic.

## Methods

### Organoid culture and assays

#### Organoid culture.

Organoid cultures were performed as previously described (3). PDX tumors were minced with no. 22 blades into 1–2 mm fragments, then digested with 1 mg/mL collagenase/dispase (Roche) for 30–40 minutes. The digestion was stopped by adding an equal volume of 1% BSA in DMEM, then centrifuged at 460*g* for 5 minutes. Pellets were further digested with Accutase (MilliporeSigma) for 30 minutes, then collected by centrifugation at 460*g* for 5 minutes. Pellets were then resuspended in organoid growth medium containing 10 μM Y-27632, 5% Matrigel, and supplements: 0.5 μg/mL hydrocortisone, 10 μg/mL insulin, 10 ng/mL IGF-1, 25 ng/mL FGF2, 5 ng/mL EGF, and 1% B27. The suspension was seeded onto 6-well plates precoated with Matrigel. Culture media were replaced every 4 days. WNT-containing media were provided by Andrew Aguirre (Dana Farber Cancer Institute, Boston, Massachusetts, USA) as used in Boj et al. (4).

#### Drug treatment assay.

Established organoid cultures were collected and digested as above. For organoids hard to dissociate for single cells, TrypLE (Invitrogen, Thermo Fisher Scientific) was used in place of Accutase (MilliporeSigma). Cells were diluted in organoid growth media at the density of 50,000 cells/mL, and 100 μL of the suspension was added into each well of a 96-well plate precoated with Matrigel. After 4 days of growth, media were replaced with fresh media, and drugs were dispensed using a Tecan D300e digital dispenser. Cell death was measured after 4 days using CytoTox-Glo (Promega). Organoids between passage 4 and passage 8 were used for drug assays.

#### Morphological and histological analysis.

Organoids were plated at a density of 25,000 cells/well, and images were taken every day for 12 days. About 200 images were obtained for each line. The images were analyzed for changes using the OrganoSeg software program (47) as detailed in Supplemental Methods.

### Drug treatments on PDX models

#### Establishment of xenografts.

Foxn1/Nu male mice, 4 to 6 weeks old, were purchased from Taconic and used for these studies. PDX tumors were from an in-house collection. Tumor initiation and expansion was performed as outlined in Supplemental Methods.

#### Treatment protocol.

Xenografts from experimental PDX cohorts were grown to a size of 200–250 mm^3^, at which time mice were randomized and enrolled in the study. The dose and schedule of treatments are described in the supplemental materials (Supplemental Figure 3A). Mice were treated for 28 days and monitored daily for signs of toxicity, with weights and tumor measurements taken 3 times per week. Tumor length and width were measured using a digital caliper, and the tumor volumes were estimated using the following formula: tumor volume = (length × width^2^)/2 (48). Relative TGI was calculated by the relative tumor growth of treated mice divided by the relative tumor growth of control mice (T/C). Experiments were terminated on day 28.

### Glycomic analysis

#### Preparation of N-glycans from cells or tissue.

Five million cells or 50 mg of tissue sample was used as starting material and lyophilized. After lyophilization, the samples were resuspended in 1 mL of 500 μg/mL TPCK-treated trypsin (MilliporeSigma) solution and incubated at 37°C overnight. The trypsin-digested samples were then loaded onto the columns before being washed with 6 mL of 5% acetic acid. Peptides were eluted with 2 mL of 20% 1-propanol, then 2 mL of 40% 1-propanol followed by 2 mL of 100% 1-propanol. The lyophilized peptides were resuspended in 200 μL of 50 mM ammonium bicarbonate, to which 3 μL of PNGase F (New England Biolabs) was added for a 4 hour incubation at 37°C. The PNGase F–treated samples were loaded onto the columns before being washed with 6 mL of 5% of acetic acid. Flow-through and wash fraction containing the released N-glycans were collected, pooled, and lyophilized and were ready for permethylation. If O-glycans were to be analyzed, the PNGase F–treated glycopeptides were eluted from the column with 2 mL of 20% 1-propanol, then 2 mL of 40% 1-propanol, and then 2 mL of 100% 1-propanol. Fractions were pooled and lyophilized and were ready for O-glycan preparation. For more details please refer to Supplemental Methods.

#### Permethylation of glycans (N-glycans).

Permethylation of N-glycans was carried out to increase the sensitivity of MS analysis and performed as outlined in Supplemental Methods.

#### Data acquisition/analysis.

MS data were acquired on a Bruker UltraFlex II MALDI-TOF Mass Spectrometer instrument. The reflective positive mode was used and data recorded between 500 *m/z* and 6000 *m/z* for N-glycans and between 0 *m/z* and 4000 *m/z* for O-glycans. MS profiles represent the aggregation of at least 20,000 laser shots. Mass peaks were then annotated and assigned to N-/O-glycan composition when a match was found. MS data were further analyzed and processed with mMass (49).

### Analysis of protein markers in EVs

#### Protein extraction and processing.

Passage 8–10 organoids were grown for 8 days in media supplemented with Matrigel, washed twice with DMEM, then incubated with Matrigel-free culture media for 4 days. At the end of the 4-day incubation, Matrigel-free media were collected for EV analysis. For EV protein extraction, serum-free conditioned media were passed through a 0.22 μm filter to remove any floating cells, debris, and large EVs (> ~250 nm). This cleared suspension was then passed through a 100,000 MW cutoff centrifugal concentrator device to enrich “EV” fraction from soluble free proteins. The retentate was resuspended in 500 μL of PBS and incubated with 0.5 volume (i.e., 250 μL) of Total Exosome Isolation Reagent (Thermo Fisher Scientific) followed by centrifugation at 10,000*g* to precipitate exosomes. EV pellets were solubilized in GuHCl lysis buffer [6 M GuHCl, 100 mM Tris pH 8.5, 10 mM tris(2-carboxyethyl)phosphine, 40 mM 2-chloroacetamide] and heated for 5 minutes at 95°C. Lysates were cooled on ice for 10 minutes, sonicated (Branson probe sonifier 10% duty cycle, 3 times, 20 seconds), and heated again (95°C for 5 minutes). Lysates were centrifuged for 30 minutes at 10,000*g* at 4°C, and cleared supernatant was removed to a clean tube. GuHCl concentration was diluted to less than 0.75 M using 100 mM Tris pH 8.5, and the samples were incubated overnight at 37°C with trypsin (1:50 w/w). The reaction was stopped by adding trifluoroacetic acid to a final concentration of 0.1%, and the peptides were desalted using C18 Sep-Pak cartridges.

#### Western blotting of EV markers.

EVs used in Western blotting analysis were purified from patient plasma using ExoQuick Ultra (System Biosciences, EQULTRA-20A-1), following the manufacturer’s instruction. Final elutes were dissolved in RIPA buffer (1/10 of elute volume). Signals were detected and quantitated with the LI-COR Odyssey system. To calculate marker ratios, intensities of protein bands of each marker were divided by signals of CD9 bands of the same patient. To rescale the marker ratios, the median value of each marker ratio in the whole patient cohort was set to be 100; then all other values were rescaled accordingly. Antibodies used were CD9, Cell Signaling Technology 13174; VGLUT2, Cell Signaling Technology 71555; GPC4, R&D Systems, Bio-Techne, MAB9195; MUC1, BD Biosciences 555925; EGFR, Cell Signaling Technology 4267; EPCAM, BioLegend 118201; CD44v6, Thermo Fisher Scientific BMS125; CD14, BioLegend 367101; annexin A11, Thermo Fisher Scientific MA5-25052; and sLeX, BD Biosciences 551344.

#### LC-MS/MS analysis and data analysis.

Peptides were analyzed with easy-nLC 1100 (Proxeon) coupled to Q-Exactive HF-X. Raw MS files were analyzed by MaxQuant 1.6 with the Andromeda search engine. Tandem MS spectra were searched against the “Reference proteome” of human (taxonomic ID 9606) downloaded from UniProt. The search included variable modifications of methionine oxidation and N-terminal acetylation and fixed modification of cysteine carbamidomethylation. Peptides of minimum 7 amino acids and maximum 2 missed cleavages were allowed for the analysis. False discovery rate of 1% was used for the identification of peptides and proteins. The data sets were then log transformed and quantile normalized, and statistically significant changes were determined using empirical Bayes analysis as implemented in the limma package.

#### Human blood collection and processing for EV analysis.

Clinical data and blood samples from patients with confirmed histopathologic diagnosis of PDAC, healthy controls, and patients with clinical diagnoses of benign GI diseases were obtained with an IRB-approved protocol from May 2018 to September 2018. After obtaining informed consent, whole-blood samples were collected in EDTA Vacutainer tubes (BD Biosciences). Plasma was obtained after initial centrifugation of 1300*g* for 15 minutes. Two additional centrifugations of 2500*g* for 15 minutes were performed to remove cellular debris. The remaining plasma samples were stored in aliquots at 80°C.

### Statistics

#### Statistical methods.

mRNA expression was quantified using quantitative PCR, and statistical significances were calculated using 2-tailed *t* test. *P* value less than 0.05 was considered significant. Glycan abundances in PXO and PDX were analyzed using 2-tailed *t* test, and a *P* value less than 0.05 was considered significant. EV biomarker signals between patient cohorts were analyzed using Kolmogorov-Smirnov test, and a *P* value less than 0.05 was considered significant.

Whole-exome sequencing analysis was performed as outlined in the Supplemental Methods section.

#### Drug sensitivity analysis.

Organoid cell viability after drug treatment was normalized to the average number of untreated cells. Response to drug concentrations was analyzed using weighted n-parameter logistic regression, nplr, R package (50), and the AUC was estimated using Simpson’s rule. The AUC of each PXO was compared with the TGI of PDX for comparative analysis of drug response.

All PCAs in this article were performed by a singular value decomposition of the normalized and scaled MS data using stats R package.

#### Pathway analysis.

Proteins identified in organoid secreted media were analyzed for predictions of protein interactions and their functional associations using STRING database (51), which incorporates known and predicted protein-protein interactions.

### Study approval

The animal study was conducted following a protocol approved by the Institutional Animal Care and Use Committee at Beth Israel Deaconess Medical Center. Patient sample collection and analysis were conducted following a protocol approved by an IRB at Beth Israel Deaconess Medical Center.

## Author contributions

LH conducted experiments, performed overall experimental design, optimized relevant protocols, and wrote the manuscript with SKM; BB prepared the clinical protocol, coordinated obtaining patient samples, reviewed patient information for EV studies, and performed multiple EV immunoblots; IP performed mass spectrometric analysis and data processing for proteomic and phosphoproteomic analysis; DA participated in organoid morphology analysis, sample preparation for glycomics, and drug assays; POF and AK designed and performed the olaparib single-agent treatments on organoids and PDX models and panel sequencing of Panc163; OG participated in drug assays and coordinated drug treatments on PDX models; AB participated in drug assays and sample preparation for proteomic studies; EER and SDL performed mass spectrometric analysis and data processing for glycomic studies; NP and JGC performed drug treatments on PDX mouse models; JG prepared clinical protocols for some of the patient samples used in EV studies; RG evaluated histopathological features of PXO and PDX; SPDP coordinated obtaining clinical information for PDX models; VSG and GD consulted on EV studies; MSS and SDF helped obtain clinical samples for EV studies; AK planned olaparib treatment experiments and conducted related data analysis with POF; RDC coordinated and contributed experimental design on glycomic study; AE coordinated and contributed experimental design on proteomic study; LBM performed analysis and interpretation of data from omic studies, in particular analysis, applying Jenks model to AUC values, interpretation of drug response, analyzing glycomics data, and manuscript preparation; CML worked with LBM on analysis of genomic studies; MH established PDX models and contributed to experimental design and data analysis; and SKM was responsible for the overall experimental design and coordinated collaborations, data analysis, and manuscript preparation.

## Supplementary Material

supplemental data

## Figures and Tables

**Table 1:**
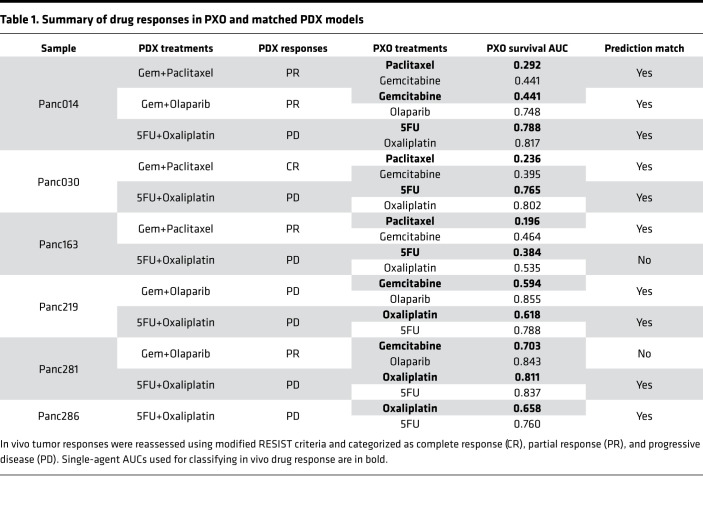
Summary of drug responses in PXO and matched PDX models

## Version 1 09/29/2020

In-Press Preview

## Version 2 11/05/2020

Electronic publication
