# Supplementary material for: PDX-derived organoids model in vivo drug response and secrete biomarkers
Source: JCI Insight. 2020 Nov 5;5(21):e135544. doi: 10.1172/jci.insight.135544 (PMC7710298; doi:10.1172/jci.insight.135544)
Supplement: supplemental data [file jciinsight-5-135544-s010.pdf]

A

PATIENT				ORIGIN				TREATMENTS			
Xenograft	AGE	GENDER	Ethnic Backgroud	Tumor Type	Tumor AP	Origin	Tumor stage (at tumorgraft collection)	At time of tumorgraft collection	Neoadjuvant	Adjuvant	Mutation Load/Mb
Panc-017	69	M		Pancreas	Adenocarcinoma	Primary Tumor	pT3N0M0	None	None	None	12
Panc-030	79	F	Caucasian	Pancreas	Adenocarcinoma	Primary Tumor	Stage IV	None	Xeloda + Radiotherapy	None (exitus 2 months after surgery)	13
Panc-163				Pancreas	Ductal Adenocarcinoma						8
Panc-219				Pancreas	Ductal Adenocarcinoma						16
Panc-265				Pancreas	Ductal Adenocarcinoma						14
Panc-281				Pancreas	Ductal Adenocarcinoma						25
Panc-286				Pancreas	Ductal Adenocarcinoma						39
Panc-014	77	F	Caucasian	Biliar	Extrahepatic biliary adenocarcinoma	Primary Tumor	pT4N1M0	None	None	None	10

B

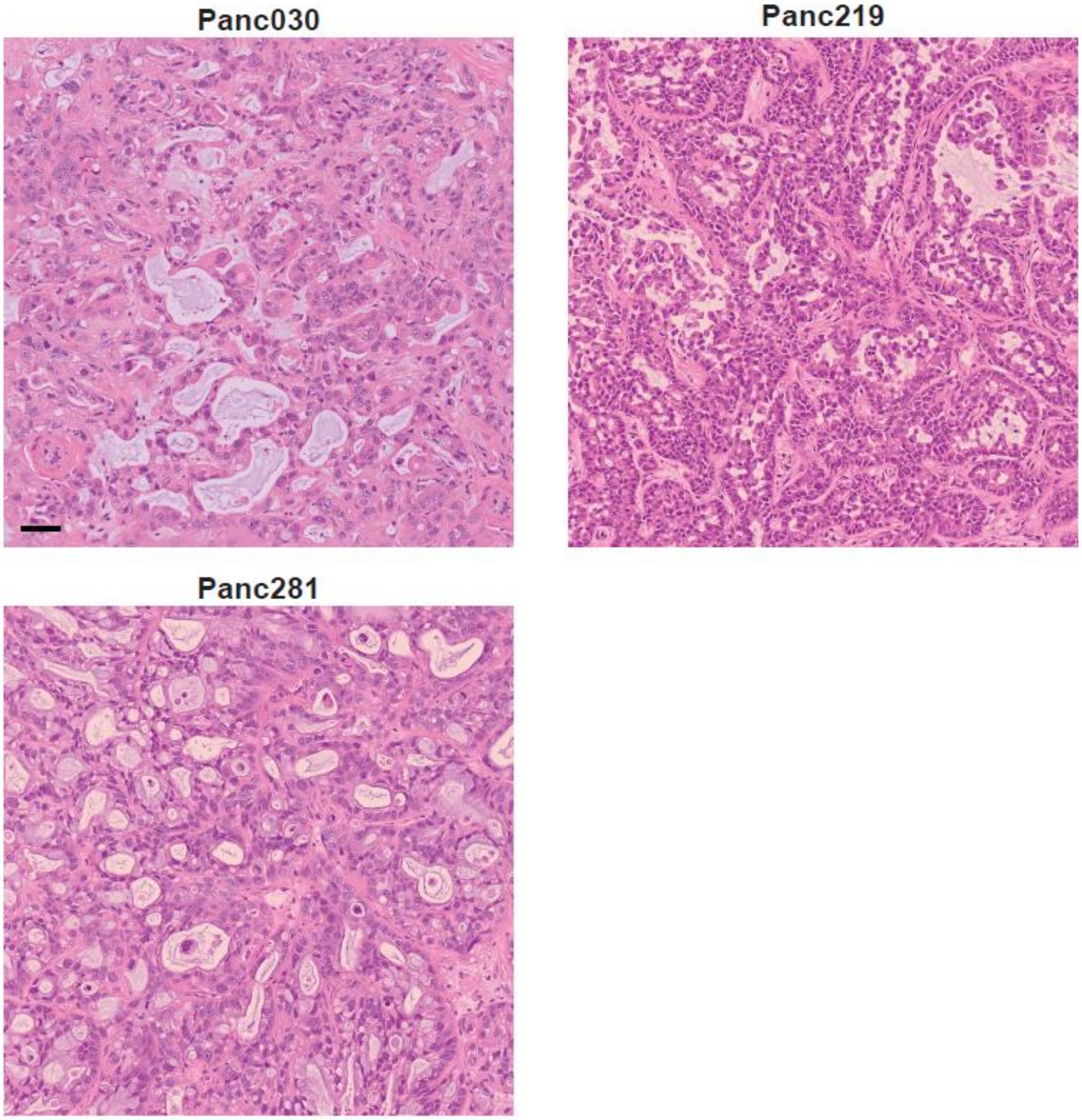

Figure S1 PDX models used in this study. (A) Information on patients whose tumors were used for establishing PDX models. **(B)** H&E images of PDX tumors with large field-of-view. Scale bar, 100 µm.

A

**Combo1**	Gemcitabine (30μM-30nM)	Olaparib (10μM-10nM)
**Combo2**	Gemcitabine (30μM-30nM)	Paclitaxel (3μM-3nM)
**Combo3**	5FU (10μM-10nM)	Oxaliplatin(10μM-10nM)

B

Name	Vehicle	Route of Adm	Dose	Treatment Duration	References
Gemcitabine	0.9% NaCl	IP injection	100 mg/kg dose every 4 days	28	Hall et al , 2016, J Transl Med
Olaparib	DMSO + PBS	IP injection	200 mg/kg dose	28	Rottemberg et al,
Paclitaxel	Cremophor diluted with 10% Ethanol	IP injection	30 mg/kg dose every 4 days	28	Fry et al, 2004, Mol Cancer Therapeutics
5-Fluouracil	Sterile 5% Dextrose in H2O	IP injection	100mg/kg dose, once a week	28	Hall et al , 2016, J Transl Med
Oxaliplatin	Sterile 5% Dextrose in H2O	IP injection	10mg/kg dose, once a week	28	Hall et al , 2016, J Transl Med

C

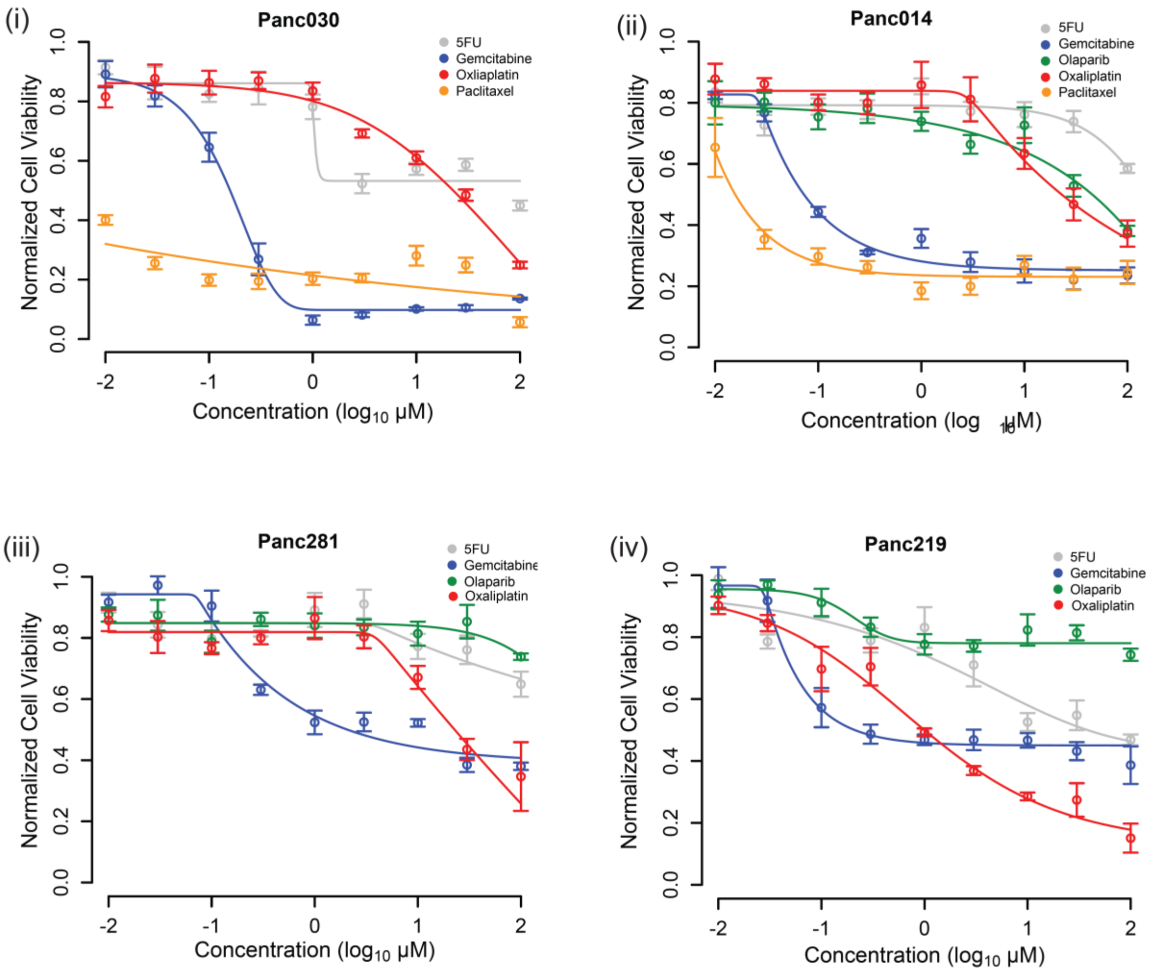

Figure S2. Information for chemotherapy treatments on PXO and PDX models (A) Information of combination treatments on PXOs **(B)** Doses and schedules for treatments on PDX mouse models. **(C)** Dose-dependent responses to single regent treatments in PXOs.

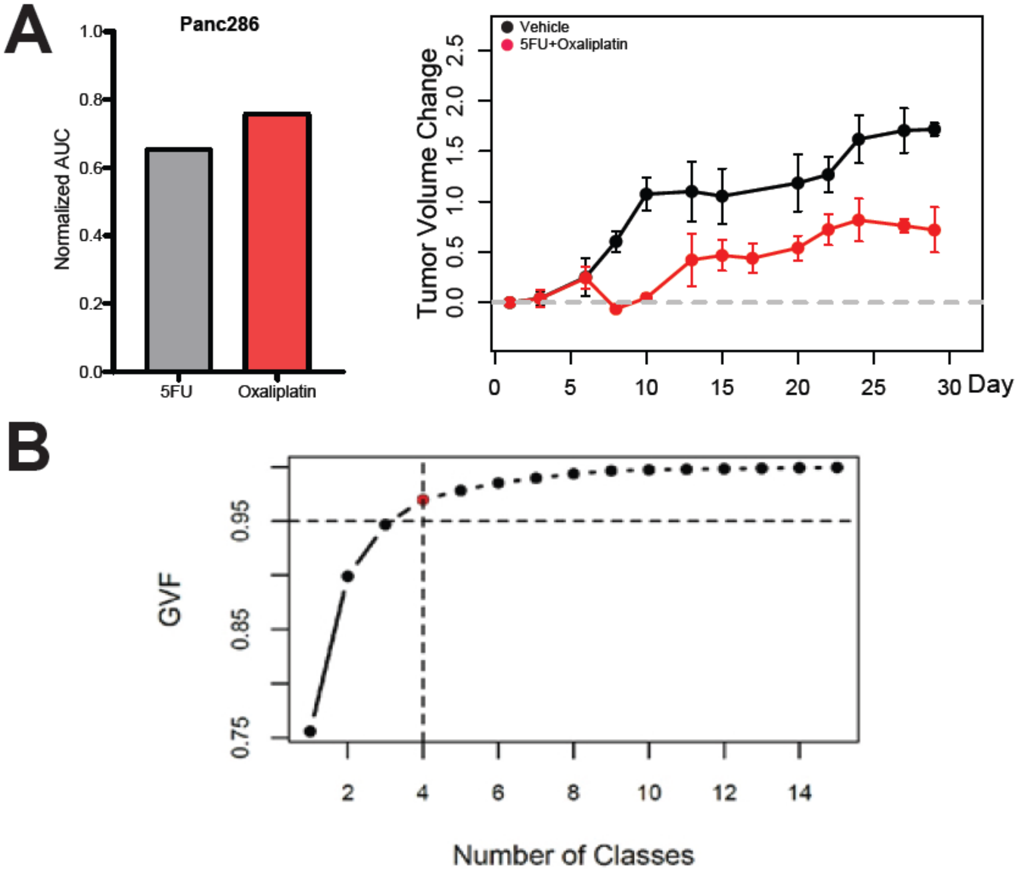

Figure S3. Comparing responses classification in vitro and in vivo (A) Treatments on Panc286 PXO and PDX models. **(B)** Goodness of fit for Jenkins break classification.

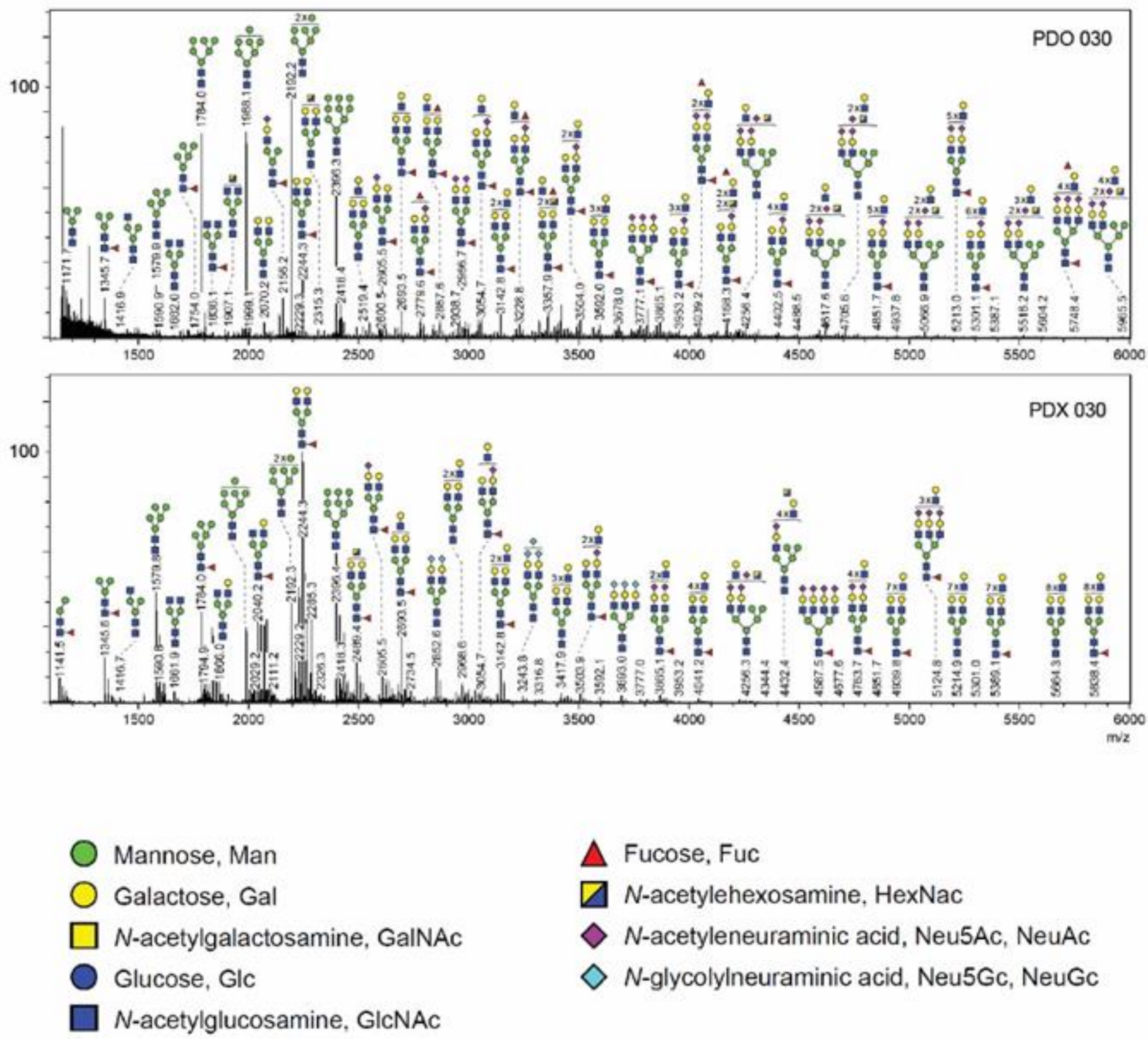

Figure S4: Mass spectrometric analysis of N-glycans in PDX and PDO of Panc030. The structures and compositions of glycans were illustrated on top of peaks of corresponding m/z values.

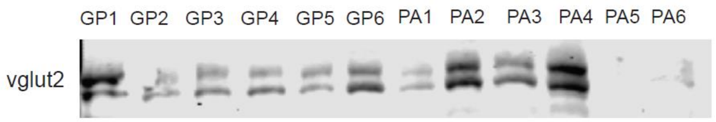

Figure S5 Immunoblot of Vglut2 proteins in EVs from patient plasma. EVs from 15 µl of plasma were input per lane.

A2M	CP	HIST1H3I	NUTF2	RSF1
AAK1	CPD	HIST1H3J	NXN	RSU1
AARS	CPE	HIST1H4A	OAF	RTCB
ABAT	CPNE1	HIST1H4B	OGA	RTRAF
ABCA7	CPOX	HIST1H4C	OGDH	RUFY3
ABHD10	CPS1	HIST1H4D	OGN	RUVBL1
ABHD14B	CPSF2	HIST1H4E	OLA1	RUVBL2
ABI1	CPSF6	HIST1H4F	OLFML3	RYR3
ACAA1	CPXM1	HIST1H4H	OPTN	S100A1
ACAA2	CRABP2	HIST1H4I	OSGEP	S100A10
ACACA	CRMP1	HIST1H4J	OSTF1	S100A11
ACAT1	CRTAP	HIST1H4K	OTUB1	S100A12
ACAT2	CRYM	HIST1H4L	OXCT1	S100A14
ACLY	CRYZ	HIST2H2AC	P3H1	S100A16
ACO1	CS	HIST2H2BE	P4HB	S100A2
ACO2	CSAD	HIST2H3A	PA2G4	S100A4
ACOT7	CSE1L	HIST2H3C	PABPC1	S100A6
ACSF2	CSH1	HIST2H3D	PACSIN2	S100A7
ACTB	CSNK2A1	HIST2H3PS2	PAFAH1B1	S100A7A
ACTC1	CSNK2B	HIST2H4A	PAFAH1B2	S100A8
ACTG1	CSRP1	HIST2H4B	PAFAH1B3	S100A9
ACTG2	CSTA	HIST4H4	PAICS	S100P
ACTN1	CSTB	HK1	PAK2	SAE1
ACTN3	CSTF2	HLA-A	PAM	SARS
ACTN4	CSTF3	HLA-B	PAPLN	SART3
ACTR1A	CTBP1	HLA-C	PAPSS1	SCFD1
ACTR2	CTGF	HMBS	PARK7	SCG2
ACTR3	CTNNA1	HMCN1	PARVA	SCG3
ADAMTS7	CTNNA2	HMGA1	PCBP1	SCG5
ADD1	CTNNB1	HMGB1	PCBP2	SCN9A
ADGRA2	CTNNBL1	HNRNPA1	PCCA	SCPEP1
ADGRG6	CTNND1	HNRNPA2B1	PCCB	SCRN1
ADH5	CTPS1	HNRNPA3	PCDHA4	SCRN3
ADK	CTSB	HNRNPC	PCDHB16	SCUBE3
ADRM1	CTSC	HNRNPD	PCDHB2	SDC4
ADSL	CTSD	HNRNPF	PCMT1	SDCBP
ADSS	CTSH	HNRNPH1	PCNA	SDF4
AEBP1	CUL3	HNRNPK	PCOLCE	SDHA
AFP	CUL4B	HNRNPM	PCSK1	SEC13

AGAP2	CUL5	HNRNPR	PCSK1N	SEC23A
AGL	CUTA	HNRNPU	PCSK2	SEC23B
AGR2	CXCL12	HNRNPUL2-BSCL2	PCSK5	SEC24C
AGRN	CYB5R3	HOOK3	PCSK9	SELENBP1
AHCY	CYFIP2	HPGD	PDCD10	SELENOF
AHCYL1	DAG1	HPRT1	PDCD4	SEMA3B
AHNAK	DARS	HPX	PDCD6	SEPHS1
AIMP1	DBT	HS6ST1	PDCD6IP	11-Sep
AIMP2	DCD	HSBP1	PDGFC	2-Sep
AIP	DCN	HSD17B4	PDGFD	6-Sep
AK1	DCTN1	HSP90AA1	PDGFRL	7-Sep
AK2	DCTN2	HSP90AB1	PDHA1	8-Sep
AKAP13	DCTN3	HSP90AB2P	PDHB	9-Sep
AKR1A1	DCXR	HSP90AB4P	PDIA3	SERPINA1
AKR1B1	DDB1	HSP90B1	PDIA4	SERPINB3
AKR1B10	DDX1	HSP90B2P	PDIA6	SERPINB4
AKR1C3	DDX39B	HSPA13	PDXK	SERPINB5
ALAS1	DDX3X	HSPA1B	PEA15	SERPINE1
ALDH16A1	DDX5	HSPA2	PEBP1	SERPINE2
ALDH18A1	DDX6	HSPA4	PFDN4	SERPINF1
ALDH1A1	DDX60L	HSPA4L	PFDN5	SERPINH1
ALDH1A2	DES	HSPA5	PFKM	SERPINI1
ALDH1L1	DHRS9	HSPA6	PFKP	SET
ALDH2	DHX15	HSPA8	PFN1	SF3A1
ALDH5A1	DHX9	HSPA9	PFN2	SF3A3
ALDH7A1	DIAPH1	HSPB1	PGAM1	SF3B1
ALDH9A1	DKK1	HSPB6	PGD	SF3B2
ALDOA	DKK3	HSPD1	PGK1	SF3B3
ALDOC	DKK4	HSPG2	PGLS	SF3B4
ALYREF	DLAT	HSPH1	PGM1	SFN
ANGPT1	DLD	HTATIP2	PGM2	SFPQ
ANGPTL4	DMBT1	HTATSF1	PGM3	SFRP1
ANP32A	DNAJA2	HTRA1	PGP	SFRP5
ANP32E	DNAJB11	HUWE1	PHGDH	SGTA
ANXA1	DNAJC10	HYOU1	PHPT1	SH3GLB1
ANXA11	DNAJC3	IARS	PICALM	SHH
ANXA2	DNAL1	IARS2	PITHD1	SIL1
ANXA3	DNM1L	IDH1	PKHD1L1	SKIV2L
ANXA4	DNM2	IDH2	PKM	SKP1
ANXA5	DNPEP	IDH3A	PLAA	SLC17A6

ANXA6	DPEP3	IDH3B	PLAT	SLC1A5
ANXA7	DPY30	IDH3G	PLAU	SLC25A5
AOC1	DPYD	IDUA	PLBD1	SLC2A1
AOX1	DPYSL2	IFI30	PLCD3	SLC3A2
AP1B1	DPYSL3	IFI35	PLCXD3	SMAD4
AP1G1	DPYSL5	IGF2	PLD3	SMARCC2
AP1M1	DRAP1	IGFALS	PLEC	SMARCE1
AP1M2	DRG2	IGFBP3	PLIN3	SMS
AP2A1	DSG1	IGFBP5	PLOD1	SNAP25
AP2A2	DSG2	IGFBP6	PLOD2	SND1
AP2B1	DSP	IGHA2	PLOD3	SNRNP200
AP2M1	DST	IGHG1	PLPBP	SNRNP70
AP3D1	DSTN	IGKC	PLS1	SNRPD1
APEX1	DUSP28	IGLC3	PLS3	SNRPD3
API5	DYNC1H1	IGSF1	PLSCR3	SNRPE
APLP2	DYNLL1	ILF2	PLTP	SNRPG
APOA1	ECHDC1	ILF3	PLXNB2	SNX1
APOC3	ECHS1	IMPDH2	PMM2	SNX6
APOE	ECM1	INA	PMPCB	SORD
APP	ECPAS	INF2	PNP	SP110
APPL1	EEA1	INHBB	POLD1	SPAG9
APRT	EEF1A1	INPP1	POP1	SPARC
ARAP1	EEF1A2	INS	POR	SPATS2L
ARCN1	EEF1B2	IPO4	POSTN	SPEN
ARF1	EEF1D	IPO5	PPA1	SPINT2
ARFIP1	EEF1E1	IPO7	PPCS	SPOCK2
ARHGAP1	EEF1G	IPO9	PPIA	SPR
ARHGDIB	EEF2	IQGAP1	PPID	SPTAN1
ARL3	EFEMP1	IRGQ	PPIP5K2	SPTB
ARL6IP5	EFEMP2	ISOC1	PPME1	SPTBN1
ARPC1A	EFTUD2	ISOC2	PPP1CB	SRI
ARPC2	EHD1	IST1	PPP1CC	SRP14
ARPC3	EHD4	ISYNA1	PPP1R12A	SRP68
ARPC4-TTLL3	EIF2S1	ITGA2	PPP1R7	SRPRA
ARPC5	EIF2S3	ITGA3	PPP2R1A	SRPX
ARPC5L	EIF3A	ITGA6	PPP2R2A	SRPX2
ARRB1	EIF3B	ITGAV	PPP2R5E	SRSF10
ASL	EIF3C	ITGB1	PPP4R3A	SRSF11
ASNA1	EIF3D	ITGB4	PRCP	SRSF3
ASNS	EIF3E	ITIH2	PRDX1	SRSF7
ASPH	EIF3F	ITIH4	PRDX2	SSB

ASS1	EIF3H	ITM2B	PRDX3	SSC5D
ATG16L1	EIF3K	ITM2C	PRDX4	SSRP1
ATG2B	EIF3L	ITPA	PRDX5	ST13
ATG4B	EIF3M	JAG1	PRDX6	ST3GAL1
ATG7	EIF4A1	JUP	PRELP	STC1
ATIC	EIF4A2	KARS	PREP	STIP1
ATP1A1	EIF4A3	KCTD12	PREX1	STK24
ATP1A3	EIF4G1	KHDRBS1	PRKACA	STOM
ATP1B1	EIF5	KHSRP	PRKACB	STXBP1
ATP5F1A	EIF5A	KIF5B	PRKAR1A	STXBP2
ATP5F1B	EIF5B	KLHL41	PRKAR2A	SUB1
ATP5MF-PTCD1	EIF6	KMT2A	PRKAR2B	SUCLA2
ATP6AP1	EML1	KPNA1	PRKD1	SUCLG2
ATP6AP2	EML2	KPNA2	PRMT1	SULF2
ATP6V0A1	ENO1	KPNA3	PRMT5	SYN1
ATP6V0D1	ENO2	KPNA4	PRPF19	SYNCRIP
ATP6V1A	ENO3	KPNA6	PRPF31	TACSTD2
ATP6V1B2	ENOPH1	KPNB1	PRPF40A	TAGLN
ATP6V1C1	EPB41L3	KRT18	PRPS1	TAGLN2
ATP6V1D	EPG5	KRT2	PRPSAP1	TALDO1
ATP6V1E1	EPHA2	KTN1	PRPSAP2	TARS
ATP6V1G1	EPHX1	L1RE1	PRRC1	TCN2
ATP6V1H	EPPK1	LAMA1	PRSS22	TCP1
ATXN10	EPRS	LAMA2	PRSS23	TF
B2M	EPS15	LAMA3	PRXL2B	TFPI
B3GAT3	EPS8	LAMA4	PSAP	TGFB1
B3GNT3	ERO1A	LAMA5	PSCA	TGFBI
B4GALT1	ETF1	LAMB1	PSMA1	TGM2
B4GAT1	ETFA	LAMB2	PSMA2	THBS3
BABAM2	ETFB	LAMB3	PSMA3	TIGAR
BAG6	EXOSC7	LAMC1	PSMA4	TIMP1
BANF1	EXT1	LAMC2	PSMA5	TIMP2
BCAS2	EXT2	LAMP1	PSMA6	TIMP3
BCAT1	EZR	LAMTOR3	PSMA7	TINAGL1
BCCIP	F3	LAP3	PSMB1	TKFC
BCKDHB	F5	LARS	PSMB2	TKT
BCL2L13	FABP5	LCN2	PSMB3	TLN1
BGN	FAM114A1	LCP1	PSMB4	TLN2
BHMT	FAM129B	LDHA	PSMB5	TMEM132A
BHMT2	FAM3C	LDHB	PSMB6	TMOD3

BIN1	FAM49B	LEFTY1	PSMB7	TMSB4X
BLVRA	FARSA	LEPR	PSMC1	TNC
BMP1	FARSB	LFNG	PSMC2	TNPO1
BMPER	FASN	LGALS1	PSMC3	TNPO3
BPIFB1	FAT1	LGALS3	PSMC4	TOLLIP
BSG	FAT2	LGALS3BP	PSMC5	TOM1
BTF3	FAT4	LGALS7	PSMC6	TOM1L2
BZW1	FBLN1	LGALS7B	PSMD1	TOMM70
BZW2	FBLN2	LGMN	PSMD11	TOP1
C11orf54	FBLN5	LIPA	PSMD12	TPD52L2
C1R	FBN1	LIPG	PSMD13	TPI1
C1S	FBN2	LMNA	PSMD14	TPM1
C1orf50	FBP1	LMNB1	PSMD2	TPM3
C3	FBXO2	LONP1	PSMD3	TPP2
C5	FBXO39	LOXL2	PSMD4	TPR
C5orf51	FCGBP	LOXL3	PSMD5	TRABD2A
CA12	FDPS	LPL	PSMD6	TRAP1
CACYBP	FERMT2	LPP	PSMD7	TRIM4
CADPS	FGB	LRBA	PSME1	TRIP10
CALM3	FGFBP1	LRP1	PSME2	TRMT1
CALML3	FGG	LRRC17	PSME3	TRMT6
CALML5	FH	LRRC59	PSMF1	TRMU
CALR	FIS1	LSM5	PSMG2	TSKU
CAMK2D	FKBP1A	LSM7	PSPC1	TSPAN6
CAND1	FKBP4	LSM8	PTBP1	TSPAN8
CAP1	FKBP5	LTA4H	PTER	TSPYL2
CAPN1	FLII	LTBP1	PTGES3	TTC13
CAPN2	FLNB	LTBP2	PTGR1	TTYH3
CAPN5	FLOT2	LUC7L3	PTGR2	TUBA1A
CAPNS1	FMOD	LUM	PTK2	TUBA1B
CAPS	FN1	LY6D	PTPA	TUBA1C
CAPZA1	FNTA	LYPLA1	PTPMT1	TUBA4A
CAPZA2	FRAS1	LYPLA2	PTPN23	TUBB
CAPZB	FREM2	LYST	PTPRF	TUBB2A
CARMIL1	FSCN1	LYZ	PTPRN	TUBB3
CASK	FSTL1	LZTFL1	PTX3	TUBB4A
CASP14	FTH1	MACROD1	PUF60	TUBB4B
CAT	FTL	MAGOHB	PURA	TUBB6
CBR1	G6PD	MAN1A1	PXDN	TUBB8
CBR3	GALE	MAN2A1	PXN	TXN
CBX3	GALNT2	MAN2B1	PXYLP1	TXNDC12

CCAR2	GANAB	MAP1B	PYCR3	TXNDC5
CCDC80	GAPDH	MAP2K2	PYGB	TXNL1
CCDC91	GAPDHS	MAPK1	PYGL	TYMP
CCDC96	GARS	MAPK3	QARS	U2AF1L5
CCT2	GART	MAPRE3	QPCT	U2AF2
CCT3	GAS6	MAT2A	QSOX1	UAP1
CCT4	GATM	MATN4	QSOX2	UBA1
CCT5	GBA	MATR3	RAB10	UBA2
CCT6A	GBE1	MBP	RAB11A	UBA5
CCT6B	GCLC	MCTS1	RAB11B	UBB
CCT7	GCNT3	MDH1	RAB14	UBE2D2
CCT8	GDF15	MDH2	RAB1A	UBE2K
CD109	GDI1	MDK	RAB1B	UBE2M
CD14	GDI2	ME2	RAB21	UBE2N
CD44	GFPT1	ME3	RAB27B	UBE2V1
CD81	GGH	MENT	RAB2A	UBE2V2
CD9	GIT1	MET	RAB39A	UCHL1
CD97	GLA	MFAP2	RAB3A	UCHL3
CDA	GLB1	MGAT5	RAB4B	UCHL5
CDC37	GLG1	MIF	RAB5A	UGDH
CDC42	GLO1	MINDY1	RAB5B	UGGT1
CDH1	GLOD4	MME	RAB5C	UGP2
CDKN2AIPNL	GLRX3	MMP1	RAB6B	USO1
CEACAM1	GLS	MMP11	RAB7A	USP14
CEACAM5	GLUD1	MMP2	RABEP1	USP47
CEACAM6	GLUL	MMP28	RABEP2	USP5
CEL	GLYR1	MMP7	RABGGTA	USP7
CEMIP2	GM2A	MPST	RABL6	VAMP2
CEP63	GMFB	MRC2	RAC1	VAMP3
CFH	GMPPA	MSLN	RACK1	VAPA
CFL1	GMPPB	MSN	RAN	VARS
CHGA	GMPR2	MTAP	RANGAP1	VAT1
CHGB	GMPS	MTHFD1	RAP1A	VAT1L
CHI3L1	GNA14	MTPN	RAP1GDS1	VCAN
CHORDC1	GNAI2	MUC1	RARRES1	VCL
CHST6	GNAI3	MUC2	RARS	VCP
CHSY3	GNAO1	MUC4	RAVER1	VGF
CIB1	GNAQ	MUC5AC	RBM39	VIL1
CKB	GNAS	MUC5B	RBP3	VIM
CKMT1A	GNB1	MVK	RBP4	VPS13C
CKMT1B	GNB2	MVP	RDX	VPS29

CLCA2	GNB3	MX1	REEP6	VPS35
CLIC1	GNG10	MXRA5	RELN	VPS37B
CLIC4	GNL1	MYH10	RHOA	VPS45
CLIC5	GNPNAT1	MYH14	RHOC	VTA1
CLPB	GORASP2	MYH9	RIC8A	VWA2
CLSTN1	GPC1	MYL12A	RNH1	WARS
CLTA	GPC6	MYL6	RO60	WASF2
CLTC	GPD1L	MYO6	RPA2	WDR1
CLTCL1	GPHN	NAE1	RPL10	WDR61
CLU	GPR12	NAMPT	RPL12	WNT5A
CMAS	GPS1	NANS	RPL13A	XDH
CMPK1	GPX1	NAP1L1	RPL15	XPNPEP1
CNDP2	GRHPR	NAP1L4	RPL17	XPO1
CNMD	GRN	NAPA	RPL18	XRCC5
CNN1	GSDMA	NAPRT	RPL18A	XRCC6
CNN3	GSN	NARS	RPL21	YARS
CNOT1	GSPT1	NCAM1	RPL22	YKT6
CNP	GSTK1	NCKAP1	RPL23	YWHAB
CNPY2	GSTM1	NCL	RPL27	YWHAE
CNTN1	GSTM2	NDFIP1	RPL27A	YWHAG
COCH	GSTO1	NDNF	RPL3	YWHAH
COG3	GSTP1	NDRG1	RPL38	YWHAQ
COL12A1	GSTT1	NDRG2	RPL4	YWHAZ
COL14A1	GSTZ1	NEFM	RPL5	ZBTB11
COL18A1	GTF2I	NEO1	RPL6	ZNF326
COL1A1	GYG1	NGDN	RPL7	ZPR1
COL1A2	H2AFV	NID1	RPL7A	
COL3A1	H2AFY	NID2	RPLP0	
COL4A1	H3F3B	NIT1	RPLP1	
COL4A2	HABP2	NME1-NME2	RPLP2	
COL5A1	HADH	NMNAT1	RPN2	
COL5A2	HADHA	NMU	RPRD1B	
COL6A2	HARS	NOL3	RPS10	
COL6A3	HBA1	NOLC1	RPS11	
COL7A1	HBA2	NONO	RPS12	
COMMD3-BMI1	HBB	NOP58	RPS13	
COMMD9	HBE1	NPC2	RPS15	
COMT	HBG2	NPEPPS	RPS15A	
COPA	HCFC1	NPM1	RPS16	
COPB1	HDAC2	NPM3	RPS17	

COPB2	HDHD2	NPNT	RPS18	
COPE	HEBP1	NPTN	RPS19	
COPG1	HIBADH	NPTX1	RPS2	
COPG2	HINT1	NPTX2	RPS20	
COPS2	HINT2	NPW	RPS21	
COPS3	HIST1H1B	NRBP1	RPS23	
COPS4	HIST1H1E	NRP1	RPS24	
COPS5	HIST1H2AC	NSD1	RPS25	
COPS6	HIST1H2AJ	NSF	RPS3	
COPS7A	HIST1H2BN	NT5C2	RPS3A	
COPS7B	HIST1H3A	NT5E	RPS4X	
COPS8	HIST1H3B	NTN1	RPS5	
COPS9	HIST1H3C	NTS	RPS6	
COPZ1	HIST1H3D	NUCB1	RPS7	
CORO1A	HIST1H3E	NUDC	RPS9	
CORO1B	HIST1H3F	NUDT21	RPSA	
CORO1C	HIST1H3G	NUMA1	RRAS	
CORO7-PAM16	HIST1H3H	NUP155	RRBP1	

Table S1 Proteins identified in tumor organoid secreted extracellular vesicles

	Age	Gender	Diagnosis	CA19-9	NCCN Stage	Treatment
**Pancreatic Cancer**						
PA1	59	M	PDAC	71	3	naïve
PA2	37	F	PDAC	33	3	naïve
PA3	70	F	PDAC	6707	4	naïve
PA4	67	F	PDAC	10	2	naïve
PA5	75	F	PDAC	213	1	naïve
PA6	63	M	PDAC	3	1	naïve
PA7	71	F	PDAC	577	2	naïve
PA8	66	F	PDAC	15273	4	naïve
PA9	51	M	PDAC	2378	3	naïve
PA10	65	F	PDAC	4139	3	naïve
PA11	59	F	PDAC	38	4	naïve
PA12	58	F	PDAC	175	3	naïve
PA13	79	F	PDAC	554	2	naïve
PA14	71	M	PDAC	3	1	naïve
PA15	80	F	PDAC	70,000	4	naïve

	Age	Gender	Diagnosis	CA19-9
**Benign GI Diseases**				
GP1	57	M	H.Pylori Gastritis	*N/A*
GP2	25	F	IBS	*N/A*
GP3	81	M	Liver Steatosis	*N/A*
GP4	47	M	Abdominal Pain	*N/A*
GP5	39	M	GERD	*N/A*
GP6	58	F	Gastritis	*N/A*
**Benign Pancreatic Diseases**				
CP1	86	M	Chronic Pancreatitis	*N/A*
CP2	45	M		*N/A*
CP3	77	M		*N/A*
CP4	53	M		*N/A*
CP5	77	M		*N/A*
IPMN1	72	F	IPMN	*N/A*
IPMN2	74	F		*N/A*
IPMN3	37	F		*N/A*
IPMN4	76	M		*N/A*
IPMN5	67	F		*N/A*

Table S2 Information of patients in EV study. PDAC, pancreatic ductal adenocarcinoma; GERD: Gastroesophageal reflux disease; N/A: not available; IBS: irritable bowel syndrome. Both CA19-9 levels and National comprehensive cancer network (NCCN) stage of the PDAC are shown.

Methods

Drug Treatment Assay: Established organoid cultures were collected and digested as above. For organoids hard to dissociate for single cells, TrypLE was used in place of Accutase. Cells were diluted in organoid growth media at the density of 50,000 cells/ml and 100 ul of the suspension was added into each well of 96 well pre-coated with matrigel. After 4 days of growth, media were replaced with fresh media, and drugs were dispensed using a Tecan D300e digital dispenser. Cell death was measured after 4 days using CytoTox Glo (Promega).

Morphological and Histological Analysis: Organoids were plated at a density of 25,000 cells/well and images were taken every day for 12 days. About 200 images were obtained for each line. The images were analyzed for changes using the organoseg software program [11]. Briefly, raw images were segmented and analyzed using inbuilt parameters such as area, perimeter, and eccentricity. The data were plotted as box plots using Prism. To generate organoid tissue sections, they were grown in chamber slides and fixed in 4% PFA for 2 hours followed by incubation with Hematoxylin solution for 10 minutes and washed twice with water. The organoids were scraped and sandwiched between two layers of Histogel (Sigma) using a cryomold and transferred to a tissue cassette followed by fixation in 10% formalin.

Establishment of xenografts: Four to six-week-old Foxn1/Nu mice were purchased from Taconic and utilized for these studies. All animal work carried out was approved by the BIDMC Institutional Animal Use and Care Committee (IACUC) and animals were maintained in accordance with guidelines of the American Association of Laboratory Animal Care. To initiate propagation, cryopreserved xenografts were rapidly thawed cut into ~3 x 3 x 3 mm fragments, and subsequently implanted subcutaneously in cohorts of 10 mice per PDX line studied, with one small fragments in each mouse. When tumors reached a size of 1500 mm3 they were excised for cohort expansion, cut into ~3 x 3 x 3 mm fragments, and transplanted to the final cohort of mice to be treated with relevant therapeutic agents, with bilateral subcutaneously implantation of fragments in each mouse.

Treatment protocol: Xenografts from experimental PDX cohorts were grown to a size of 200-250 mm^3, at which time mice were randomized and enrolled on the study. The dose and schedule of treatments were described in the supplemental materials (**Fig.S3A**). Mice were treated for 28 days and monitored daily for signs of toxicity, with weights and tumor measurements taken three times per week. Tumor length and width were measured using a digital caliper and the tumor volumes estimated using the following formula: tumor volume = [length x $width^2$] / 2 [30]. Relative tumor growth inhibition (TGI) was calculated by the relative tumor growth of treated mice divided by the relative tumor growth of control mice (T/C). Experiments were terminated on day 28. For evaluation of drug responses in PDX models, the RECIST calculation from Jackson Lab was used. Briefly, the percentage of tumor volume change was calculated as (Ve-Vs)/Vs*100, Ve was tumor volume at the endpoint and Vs was tumor volume at the start of treatments. The categories of responses were defined as the following: where Vm is the minimal volume change, and Va is the average volume change.

RECIST Category	Best Response: Vm	Average Response: Va
Complete Response (CR)	< -95%	< -40%
Partial Response (PR)	< -50%	<-20%
Stable Disease (SD)	< 35%	< 30%
Progressive Disease (PD)	Anything else	

Genomic concordance between PDX and PXO: Variant calls on whole exome sequencing data were made using a standard pipeline. The sequence reads were aligned in hg19 using BWA (v.0.7.8), recalibrated, and identified variants using Picard-tools/GATK v3.0. Copy number analysis on PDX-WES data was done using CNVkit and variants were filtered and annotated using Annovar for gene annotation, mutation consequences, prediction of deleteriousness, and allele frequency. SNV concordance between tumor-organoid pairs was determined from the overlap of variant calls and variant allelic fractions. For each SNV called in PDX models, we used BCFTools-v1.9 to read evidence for the SNV present in WES-organoids after filtering for base quality and mapping quality to be considered concordant. Visualization of DNA aberrations is displayed using Oncoprint/ComplexHeatmap – R package.

Drug response concordance between PDX and PXO: AUC values from PXO drug-response curve is classified into 4 classes using Jenks natural breaks classification. We used the implementation of Jenks algorithm by BAMMtools, an R package (Rabosky, et al, 2014). The optimal number of breaks was estimated the number of breaks that would give >95% goodness of variance fir (GVF).

Reference

BAMMtools: an R package for the analysis of evolutionary dynamics on phylogenetic trees, Daniel L Rabosky, Michael Grundler, Carlos Anderson, Pascal Title, Jeff J. Shi, Joseph W. Brown, Huateng Huang, Joanna G Larson, Methods in Ecology and Evolution 2014, 5, 701-707
